# An advanced sequence clustering and designation workflow reveals the enzootic maintenance of a dominant West Nile virus subclade in Germany

**DOI:** 10.1093/ve/vead013

**Published:** 2023-03-17

**Authors:** Pauline Dianne Santos, Anne Günther, Markus Keller, Timo Homeier-Bachmann, Martin H Groschup, Martin Beer, Dirk Höper, Ute Ziegler

**Affiliations:** Friedrich-Loeffler-Institut, Federal Research Institute for Animal Health, Institute of Diagnostic Virology, 17493, Greifswald-Insel Riems, Germany; Friedrich-Loeffler-Institut, Federal Research Institute for Animal Health, Institute of Diagnostic Virology, 17493, Greifswald-Insel Riems, Germany; Friedrich-Loeffler-Institut, Federal Research Institute for Animal Health, Institute of Novel and Emerging Infectious Diseases, 17493, Greifswald-Insel Riems, Germany; Friedrich-Loeffler-Institut, Federal Research Institute for Animal Health, Institute of Epidemiology, 17493, Greifswald-Insel Riems, Germany; Friedrich-Loeffler-Institut, Federal Research Institute for Animal Health, Institute of Novel and Emerging Infectious Diseases, 17493, Greifswald-Insel Riems, Germany; German Centre for Infection Research, Partner site Hamburg-Lübeck-Borstel-Riems, 17493, Greifswald-Insel Riems, Germany; Friedrich-Loeffler-Institut, Federal Research Institute for Animal Health, Institute of Diagnostic Virology, 17493, Greifswald-Insel Riems, Germany; Friedrich-Loeffler-Institut, Federal Research Institute for Animal Health, Institute of Diagnostic Virology, 17493, Greifswald-Insel Riems, Germany; Friedrich-Loeffler-Institut, Federal Research Institute for Animal Health, Institute of Novel and Emerging Infectious Diseases, 17493, Greifswald-Insel Riems, Germany; German Centre for Infection Research, Partner site Hamburg-Lübeck-Borstel-Riems, 17493, Greifswald-Insel Riems, Germany

**Keywords:** West Nile virus Germany, objective sequence clustering, virus sub species nomenclature

## Abstract

West Nile virus (WNV) is the most widespread arthropod-borne (arbo) virus and the primary cause of arboviral encephalitis globally. Members of WNV species genetically diverged and are classified into different hierarchical groups below species rank. However, the demarcation criteria for allocating WNV sequences into these groups remain individual and inconsistent, and the use of names for different levels of the hierarchical levels is unstructured. In order to have an objective and comprehensible grouping of WNV sequences, we developed an advanced grouping workflow using the ‘affinity propagation clustering’ algorithm and newly included the ‘agglomerative hierarchical clustering’ algorithm for the allocation of WNV sequences into different groups below species rank. In addition, we propose to use a fixed set of terms for the hierarchical naming of WNV below species level and a clear decimal numbering system to label the determined groups. For validation, we applied the refined workflow to WNV sequences that have been previously grouped into various lineages, clades, and clusters in other studies. Although our workflow regrouped some WNV sequences, overall, it generally corresponds with previous groupings. We employed our novel approach to the sequences from the WNV circulation in Germany 2020, primarily from WNV-infected birds and horses. Besides two newly defined minor (sub)clusters comprising only three sequences each, Subcluster 2.5.3.4.3c was the predominant WNV sequence group detected in Germany from 2018 to 2020. This predominant subcluster was also associated with at least five human WNV infections in 2019–20. In summary, our analyses imply that the genetic diversity of the WNV population in Germany is shaped by enzootic maintenance of the dominant WNV subcluster accompanied by sporadic incursions of other rare clusters and subclusters. Moreover, we show that our refined approach for sequence grouping yields meaningful results. Although we primarily aimed at a more detailed WNV classification, the presented workflow can also be applied to the objective genotyping of other virus species.

## 1. Introduction

Like other members of the genus *Flavivirus*, West Nile virus (WNV) has become a serious emerging zoonotic threat in Europe within the last decades (European Centre for Disease Prevention and Control 2021; Kuno et al. 1998). The first known case of WNV infection was reported in Uganda, Africa, in 1937 (Smithburn et al. 1940; Bardos et al. 1959). In the 1960s, the first occurrence of WNV in Europe was recognized due to neurological disorders in wild and domestic horses in France (Murgue et al. 2001). Around 30 years later, WNV caused the first severe outbreak of West Nile fever and West Nile neuroinvasive disease in humans in Romania (Tsai et al. 1998; Savage et al. 1999). Since then, WNV has successfully established in various countries. Southern and Eastern European countries were primarily affected by recurring WNV infections in humans, birds, and horses. The highest WNV activity in Europe was recorded in 2018 (European Centre for Disease Prevention and Control 2019; Camp and Nowotny 2020). Almost 90 per cent of all locally acquired WNV human infections in Europe, with 166 fatal cases, were reported in Italy, Greece, and Romania (European Centre for Disease Prevention and Control 2019). In parallel to this large-scale epidemic in 2018, WNV RNA–positive birds and horses were confirmed for the first time in Germany (Ziegler et al. 2019). In 2019, a significant increase in WNV cases in birds and horses and the first five autochthonous WNV human infections in Germany were reported (Robert-Koch-Institut 2020; Ziegler et al. 2020). All prerequisites for endemic WNV circulation in Germany are fulfilled, including the proven vector competence of local mosquito populations (Holicki et al. 2020) and the detection of WNV genome–positive mosquito pools (Kampen et al. 2020; Ziegler et al. 2020).

WNV has a diverse host range and is widely distributed. Accordingly, members of this species are genetically diverse, allowing for the further subgrouping within the species. However, since the International Committee on Taxonomy of Viruses confines its responsibility to the designation and demarcation of viruses from realm to species ranks (Simmonds et al. 2017; ICTV 2020), neither a standard definition of criteria for subgrouping below the species rank nor defined designations for subgroups and their hierarchical arrangement exist. Therefore, designations for hierarchical ranks (e.g. clade, cluster, subtype, and genotype) are often used inconsistently and interchangeably, leading to misunderstandings and uncertainties, as more and more whole genomes of WNV are generated. Due to its aforementioned genetic diversity, up to nine lineages have been proposed for the species *West Nile virus* (Pachler et al. 2014; Fall et al. 2017; Mencattelli et al. 2022). The designation ‘lineage’ is mostly based on monophyletic clustering of partial genome or whole-genome WNV sequences in phylogenetic analyses (Fall et al. 2017; Perez-Ramirez et al. 2017). However, the lineage classification of WNV strains remains controversial (Perez-Ramirez et al. 2017). Further subgrouping within the lineages is conducted to organize viruses into a hierarchical system comprising various arbitrarily defined and designated groups. In particular, within and between members of WNV Lineage 1 and WNV Lineage 2 (WL2), the designations are used inconsistently. Groups are usually defined based on branching into monophyletic groups from a common ancestor, and members of groups may share common characteristics such as unique and fixed amino acid (aa) substitutions (Davis et al. 2005; May et al. 2011; Anez et al. 2013; McMullen et al. 2013; Barzon et al. 2015; Di Giallonardo et al. 2016; Chaintoutis et al. 2019; Hadfield et al. 2019; Ziegler et al. 2020). Monophyletic groups other than lineages are typically labeled using a letter, region of origin, or abbreviation of the region of origin (McMullen et al. 2013; Kolodziejek et al. 2014; Ravagnan et al. 2015; Fall et al. 2017; Zehender et al. 2017; Bialosuknia et al. 2019; Ziegler et al. 2019, 2020; Bialosuknia et al. 2022). Noteworthy, nomenclatures based on geographic origin may be misleading, if not stigmatizing certain geographic regions associated or assumed with a virus origin as recently discussed for the monkeypox outbreaks in Europe in 2022 (Happi et al. 2022; Taylor 2022). For instance, a WNV sequence from Italy branched with Eastern European WL2 sequences was detected in Romania and Russia (Ravagnan et al. 2015; Bakonyi and Haussig 2020; Sikkema et al. 2020; Ziegler et al. 2020). Moreover, Ziegler et al. (2020) mentioned in the study of the 2018–19 WNV epidemic in Germany that the label ‘Eastern German WNV Clade (EGC)’, designated to a group of WNV sequences from Germany, may not be a suitable designation because ‘the EGC can have developed in the wider Southeastern and Central European hemisphere and may have been translocated only later to Eastern Germany’. Hence, labels based on geographic origin may not suit the expanding geographic or undiscovered range of a WNV sequence group.

The described situation emphasizes the need for a systematic nomenclature and objective grouping of WNV sequences into hierarchical groups below the species rank. To subdivide WNV, we further developed the objective clustering workflow established by Fischer et al. (2018) who utilized the affinity propagation clustering (APC) algorithm (Frey and Dueck 2007) as implemented by Bodenhofer, Kothmeier, and Hochreiter (2011). However, Fischer and colleagues found limitations of APC, especially for the definition of the best-suited number of clusters and therefore ultimately the definition of groups corresponding with phylogenetic analyses. To solve these issues, we refined the method to define a suitable number of groups while also incorporating agglomerative hierarchical clustering (AHC) (Bodenhofer, Kothmeier, and Hochreiter 2011) to address grouping of sequences into multiple hierarchical levels. In addition, we suggest a decimal numbering system for the hierarchical groups designated with the proposed unified and consistent labels within the WNV species. Finally, we provide an update on the WNV situation in birds and horses in Germany 2020 by applying the improved clustering workflow and our novel generic and consistent nomenclature.

## 2. Material and Methods

### 2.1 WNV screening of birds and horses

WNV infection in birds and horses is a notifiable animal disease in Germany. Cases are confirmed by real-time quantitative polymerase chain reaction (RT-qPCR) and/or the identification of WNV-specific Immunoglobulin M (IgM) in non-vaccinated horses by enzyme-linked immunosorbent assay (i.e. detection of a recent WNV infection). The samples analyzed in this study are a subset of samples from all over Germany examined for WNV by molecular or serological diagnostics (for a comprehensive overview of the WNV situation in Germany, please see Bergmann et al. 2022; Ganzenberg et al. 2022; Ziegler et al. 2022). They were derived from birds or horses (e.g. complete animals, organ samples, blood samples, and/or total RNA) tested positive by the regional veterinary laboratories of the German federal states and were subsequently sent to the national reference laboratory for WNV at the Friedrich-Loeffler-Institut (FLI), Isle of Riems, Germany, for confirmation. In addition, members of the nationwide wild bird surveillance program (for details on the members, see Ziegler et al. 2022) sent in samples from live birds or animals found dead with unknown WNV status. The nationwide wild bird surveillance program in Germany was established as a response to the first Usutu virus epizootic in 2011. This monitoring program became reputable also for the early detection of other zoonotic arboviruses, such as Sindbis virus and WNV.

### 2.2 Ethical statement

Bird clinics, veterinarians, wild bird rescue centers, and zoos provided bird carcasses for necropsy. In Germany, no specific permits are required to examine dead birds that have been submitted for necropsy. Horse clinics and veterinarians from the regional veterinary laboratories provided horse tissue samples collected in post-mortem examinations by pathological institutions. Residual blood material was available for one case originating from a WNV-infected bird, collected primarily for diagnostic purposes and for specific treatment and prognosis.

### 2.3 RNA extraction and RT-qPCR

Total RNA was extracted from tissue samples (brain, spleen, liver, spinal cord, and/or kidney) and frozen (−70°C) coagulated blood samples (cruor). For the first RNA extraction, we applied the RNeasy Mini Kit (QIAGEN) according to the manufacturer’s instructions, followed by screenings for both WNV Lineage 1 and WL2 genomes using a RT-qPCR assay (Eiden et al. 2010).

### 2.4 Whole-genome sequencing

To cover areas with and without previous WNV cases, WNV RNA–positive samples from 2020 (Table 1) were selected for whole-genome sequencing (WGS) primarily based on their geographical location and C_q_ values. In addition, samples from captive birds, wild birds, and horses from similar regions were included. These selected samples (Table 1) were subjected to a different RNA extraction protocol to ensure the acquisition of high-quality starting material for WGS. Briefly, each organ homogenate suspension (250 µl) was lysed in 750-µl TRIzol™ LS Reagent (Invitrogen), or approximately 30-mg tissue material was homogenized in 1-ml TRIzol™ reagent via TissueLyser II (QIAGEN) with a 5-mm steel bead for 2 min at 30 Hz. After phase separation, the aqueous phase was processed using the Agencourt® RNAdvance Tissue kit (Beckman Coulter) and the KingFisher Flex system (Thermo Fisher Scientific) according to the manufacturer’s instructions.

**Table 1.** Overview of WNV cases analyzed in this study. Sample numbers are used in Figs 4 and 7.

Sample no.	Sample ID	Library ID	Sequence accession	Common English name	Scientific name	Collection date	Federal state^a^
1	167/20	lib04566	OX442347	Blue tit	*Cyanistes caeruleus*	7 July 2020	BE
2	174/20	lib04567	OX442284	Snowy owl	*Bubo scandiacus*	12 July 2020	TH
3	192/20	lib04568	OX442304	Snowy owl	*Bubo scandiacus*	15 August 2020	TH
4	193/20	lib04569	OX442279	Northern goshawk	*Accipiter gentilis*	30 July 2020	SN
5	200/20	lib04570	OX442299	Bohemian waxwing	*Bombycilla garrulus*	11 August 2020	BB
6	203/20	lib04571	OX442309	Little owl	*Athene noctua*	18 August 2020	BB
7	206/20	lib04572	OX442306	Northern goshawk	*Accipiter gentilis*	8 August 2020	BE
8	214/20	lib04573	OX442308	Unspecified flamingo	*Phoenicopterus* sp.	19 August 2020	TH
9	218/20	lib04574	OX442287	Unspecified flamingo	*Phoenicopterus* sp.	18 August 2020	ST
10	339/20	lib04575	OX442293	Chilean flamingo	*Phoenicopterus chilensis*	25 August 2020	BE
11	252/20	lib04576	OX442301	Eurasian jay	*Garrulus glandarius*	August/September 2020	TH
12	283/20	lib04577	OX442291	Snowy owl	*Bubo scandiacus*	2 September 2020	ST
13	207/20	lib04717	OX442300	Northern goshawk	*Accipiter gentilis*	9 August 2020	BE
14	208/20	lib04718	OX442294	Northern goshawk	*Accipiter gentilis*	14 August 2020	BB
15	211/20	lib04719	OX442296	Blue tit	*Cyanistes caeruleus*	August 2020	BE
16	194/20	lib04720	OX442303	Blue tit	*Cyanistes caeruleus*	August 2020	SN
17	268/20 Nr. 1	lib04721	OX442295	Little owl	*Athene noctua*	September 2020	BB
18	311/20	lib04722	OX442298	Northern goshawk	*Accipiter gentilis*	September/October 2020	BE
19	314/20	lib04723	OX442348	Northern goshawk	*Accipiter gentilis*	September/October 2020	BE
20	315/20	lib04724	OX442302	Northern goshawk	*Accipiter gentilis*	September/October 2020	BE
21	224/20	lib04725	OX442283	Blue tit	*Cyanistes caeruleus*	August 2020	BE
22	282/20	lib04726	OX442277	Snowy owl	*Bubo scandiacus*	2 September 2020	ST
23	281/20	lib04727	OX442274	Unspecified flamingo	*Phoenicopterus sp.*	September 2020	ST
24	340/20	lib04728	OX442282	American flamingo	*Phoenicopterus ruber*	22 August 2020	BE
25	196/20	lib04729	OX442276	Unspecified buzzard	*Buteo* sp.	August 2020	SN
26	258/20	lib04730	OX442273	Great tit	*Parus major*	August/September 2020	SN
27	242/20	lib04731	OX442292	Northern goshawk	*Accipiter gentilis*	August 2020	BE
28	245/20	lib04732	OX442280	Unspecified flamingo	*Phoenicopterus* sp.	August 2020	BE
29	260/20	lib04733	OX442278	Domestic canary	*Serinus canaria forma domestica*	September 2020	ST
30	261/20	lib04734	OX442271	Chilean flamingo	*Phoenicopterus chilensis*	September 2020	SN
31	264/20	lib04735	OX442281	Unspecified sparrow	*Passer* sp.	September 2020	SN
32	228/20	lib04736	OX442272	Horse	*Equus caballus*	2 September 2020	SN
33	173/20	lib04737	OX442275	Alpine chough	*Pyrrhocorax graculus*	12 July 2020	ST
34	216/20	lib04738	OX442286	Blue tit	*Cyanistes caeruleus*	August 2020	TH
35	199/20	lib04739	OX442312	Northern goshawk	*Accipiter gentilis*	13 June 2020	BE
36	205/20	lib04740	OX442307	Northern goshawk	*Accipiter gentilis*	13 August 2020	BE
37	210/20	lib04741	OX442305	Northern goshawk	*Accipiter gentilis*	1 August 2020	BE
38	238/20	lib04742	OX442285	Northern goshawk	*Accipiter gentilis*	31 August 2020	BE
39	241/20	lib04743	OX442289	Northern goshawk	*Accipiter gentilis*	23 August 2020	BE
40	244/20	lib04744	OX442311	Hooded crow	*Corvus corone cornix*	18 August 2020	BE
41	219/20	lib04745	OX451204	Chilean flamingo	*Phoenicopterus chilensis*	26 August 2020	ST
42	284/20	lib04746	OX442290	Swift parrot	*Lathamus discolor*	21 September 2020	ST
43	286/20	lib04747	OX442313	Horse	*Equus caballus*	29 September 2020	ST
44	246/20	not done^b^	NA	Golden eagle	*Aquila chrysaetos*	(09 September 2020) survived	BB
45	201/20	not done^b^	NA	European greenfinch	*Carduelis chloris*	7 July 2020	BB
46	60/21	lib04758	OX442288	Blue tit	*Cyanistes caeruleus*	2020	TH
C4	115/19	lib04565	OX442297	Chinese merganser	*Mergus squamatus*	08 August 2019	BE
C5	127/18	lib04748	OX442310	Horse	*Equus caballus*	11 September 2018	BB

a Abbreviations for federal states: BE, Berlin; BB, Brandenburg; SN, Saxony; ST, Saxony-Anhalt; TH, Thuringia.

b WNV-specific multiplex PCR was unsuccessful due to low amount of WNV RNA in the sample (C_q_ 36).

WGS of WNV was performed as described by Quick et al. (2017) with some modifications. Briefly, RNA was reverse transcribed using the SuperScript™ IV First-Strand Synthesis System (Invitrogen) with random hexamers. The complementary DNA (cDNA) was subjected to the WNV-specific multiplex PCR as described by Sikkema et al. (2020). Using two different primer mixes (Table S1) and an AccuPrime™ *Taq* DNA Polymerase Kit (Invitrogen), two multiplex PCR reactions were performed. Amplicons were purified with 1.8 volume of Agencourt® AMPure XP beads (Beckman Coulter) and quantified using a NanoDrop™ ND-1000 Spectrophotometer (Thermo Fisher Scientific). These two purified and quantified amplicon pools were combined per sample in equal concentration (125 ng each) and the volume adjusted to 130 µl. Fragmentation and library preparation steps were performed according to Wylezich et al. (2018). Quantified libraries (GeneRead DNA Library L Core Kit; QIAGEN) including the Ion Torrent S5 Calibration Standard were sequenced using an Ion Torrent S5 XL instrument with Ion 530 Chip and respective reagents (Thermo Fisher Scientific) in 400-bp mode according to the manufacturer’s recommendations.

We verified the PCR-based sequencing using five WNV-positive samples from previous seasons (C1–C5; Table S2) that had already been sequenced according to the validated approach described by Wylezich et al. (2018). Two previously completed libraries of C4 and C5 were enriched for WNV using myBaits (Wylezich et al. 2018, 2021) but still only yielded partial genome sequences. On the contrary, the multiplex PCR-based approach generated complete coding sequences of all five test samples, albeit with a truncated 3´ end (23–71 nucleotides). As summarized in Table S2, the sequences from both approaches were 100 per cent identical for samples C1–C3; for samples C4 and C5, 8 and 2 substitutions were found, respectively. These results demonstrated that the multiplex PCR approach is suitable for reliable and sensitive WGS of WNV, even from samples with low WNV concentration (up to C_q_ value 31.5).

Sample No. 26 (ED-I-258/20) had a genome region with a sequencing depth lower than 30; therefore, sequencing results were confirmed with Sanger sequencing. Briefly, cDNA from Sample ED-I-258/20 was amplified using additional singleplex PCR assays (primer pairs: WNVUS1_30_LEFT and WNVUS1_30_RIGHT_2; WNVUS1_30_LEFT_2 and WNVUS1_30_RIGHT). The amplicon was sequenced with a BigDye Terminator v1.1 Cycle Sequencing kit (Applied Biosystems™, Thermo Fisher Scientific) on a 3500 Genetic Analyzer Instrument (Applied Biosystems™, Thermo Fisher Scientific).

WNV genome sequences obtained in this study were submitted to the European Nucleotide Archive under the BioProject accession number PRJEB47687.

### 2.5 Datasets

We validated our workflow using two test datasets consisting of WNV complete coding sequences previously characterized and classified into different ranks below the species level. ‘Test Dataset 1’ (TD01) consists of ninety-five WNV WGSs characterized and classified into different lineages by Fall et al. (2017). Notably, this study considered WNV Clades 1a, 1b, 1c, 4a, and 4b/9 as distinct lineages. ‘Test Dataset 2’ (TD02) consists of 150 WNV WGSs allocated to three WNV clades and six WNV Clade 1a clusters described by May et al. (2011). We also combined the sequences from these two test datasets and a sequence described as a member of the putative WNV Clade 1a Cluster 7 (Aguilera-Sepulveda et al. 2021). We referred to these sequences as ‘Test Dataset 3’ (TD03). Available complete coding sequences of WL2 and their metadata (e.g. sample collection year and country of origin) were retrieved from GenBank on 10 December 2021. WL2 dataset consisted of WNV complete coding sequences from the database and sequences acquired in this study. Accession numbers of WNV sequences per dataset are summarized in Table S3. We also prepared versions of these datasets that excluded sequences with ≥10 ambiguous nucleotides or gaps and duplicates.

### 2.6 *In-silico* analyses

#### 2.6.1 Sequence assembly

Genome sequences were assembled from raw data using the Roche/454 genome sequencer software suite v3.0 (Roche). Sequencing adapters and PCR primers were trimmed using the Newbler assembler prior to reference mapping. Initial reference-based mapping against WNV Strain 1382/2018/Berlin/Ger (MH986055.1) was done to generate a sample-specific consensus sequence. These consensus sequences were then employed as the reference for a second reference-based mapping per dataset. The resulting genome sequences were visually inspected using the Geneious Prime® 2021.0.1 software (Biomatters).

#### 2.6.2 WNV genome characterization and phylogenetic analyses

Complete coding sequences from each dataset (TD01, TD02, TD03, and WL2) were aligned using the MUSCLE algorithm (Edgar 2004) and visually inspected using Geneious Prime® 2021.0.1.

#### 2.6.3 Maximum likelihood phylogenetic analysis

The best-fitting nucleotide substitution model for each dataset was calculated using jModelTest 2.1.10 (Darriba et al. 2012). Maximum likelihood (ML) inference with the determined best substitution model and ultrafast bootstrap option (Minh, Nguyen, and von Haeseler 2013; Hoang et al. 2018) with 100,000 replicates was performed using IQ-TREE 1.6.8 (Nguyen et al. 2015). ML phylogenetic trees were viewed using FigTree software (v1.4.4, http://tree.bio.ed.ac.uk/software/figtree/).

#### 2.6.4 Bayesian phylogenetic analysis

We subjected the dataset consisting of complete genome sequences belonging to the Subclade 2.5.3 to the Bayesian Markov Chain Monte Carlo (MCMC) method implemented in the BEAST package version 1.10.4 (Drummond and Rambaut 2007; Suchard et al. 2018). We performed regression analyses of the root-to-tip genetic distance in the resulting ML trees against sampling years using TempEst (Rambaut et al. 2016). The spatiotemporal dynamics of WNV and the time to most recent common ancestors (MRCAs) were co-estimated using best-suited substitution model based on the jModelTest 2 (Darriba et al. 2012), optimal molecular clock model (relaxed uncorrelated lognormal), and best demographic scenario (the Bayesian Skygrid coalescent model), which is explained here.

The optimal molecular clock model (strict or relaxed uncorrelated log normal) and tree prior (Constant, Bayesian Gaussian Markov random field Skyride, or Bayesian Skygrid model) were selected based on the marginal likelihood estimation path sampling and stepping stone sampling methods. The MCMC chain length was run until convergence and sampled every 10^4^ iterations. Convergence was evaluated by approximating the effective sampling size (ESS) after a 10 per cent burn-in using the Tracer software version 1.7.1, with ESS values ≫200 accepted. The strength of the evidence against H_0_ was evaluated according to Kass and Raftery’s (1995) Bayes factor (BF) test as follows: BF 1–3—weak, BF 3–20—positive, BF 20–150—strong, and BF >150—very strong (comparison of each parameter is summarized in Table S4).

Phylogeographic analysis was performed using a discrete model attributing state characters represented by the detection of location (country) of each strain and the Bayesian stochastic search variable algorithm implemented in BEAST v1.10.4 (Suchard et al. 2018). TreeAnnotator v1.10.4 was employed to summarize the maximum clade credibility (MCC) tree after 10 per cent burn-in, and FigTree software v1.4.4 was utilized to visualize the MCC tree. The branches of the trees were color-coded based on the samples of geographic origin (country).

#### 2.6.5 APC-based workflow for sequence grouping

We analyzed WNV complete coding sequences using a workflow comprising the APC algorithm and AHC included in the R package ‘apcluster’ v 1.4.10 (Bodenhofer, Kothmeier, and Hochreiter 2011) implemented in R v4.1.2 (R Core Team 2022) and R studio (v2021.09.1-372). The APC algorithm requires a dissimilarity matrix as an input for clustering. For each of the determined clusters, one entity is defined as the ‘best representative’ or the ‘cluster exemplar’.

Using the Sequence Demarcation Tool (SDT_Linux64 v1.2) (Muhire, Varsani, and Martin 2014), we calculated pairwise global alignments of the coding sequences and, from these alignments, used the pairwise nucleotide identities to calculate a dissimilarity matrix by subtracting the identities from one. Subsequently, to increase the robustness and discriminatory power of the APC, these dissimilarities were squared and converted to negative values according to Fischer et al. (2018) in order to yield the suitable input data for the APC algorithm.

One major problem in clustering is finding the suitable number of clusters to subdivide the dataset into. To this end, Fischer et al. (2018) developed the ‘plateau method’ to calculate the optimum number of clusters. The number of clusters generated by APC is determined by a parameter called input preference, which by default is set to 0.5. Using the APC algorithm, the suitable ‘input preference range’ from minimum (pmin) to maximum (pmax) can be calculated. For the plateau method, the number of clusters (*z*-value) is repeatedly determined in dependence of the input preference which is increased in equal steps through the preference range. Usually, with an increase of the input preference, the number of groups monotonously increases; if a reduction occurs, this is deemed a disturbance that leads to the termination of the calculations. Fischer and colleagues defined the best-suited number of groups corresponding to the longest plateau that was observed (the same number of clusters observed consecutively for the highest number of iterations before a disturbance occurred). While in principle this was suitable, they nevertheless found that it was not optimal. Since there can be a monotonous increase of the group number without a disturbance occurring throughout the whole preference range, we tested using the last stable plateau as an alternative measure for the definition of the group number. Here, we define the last stable plateau as the last plateau without disturbance and with at least the set minimal length. For this calculation, we set the minimum number of iterations that make a plateau to three. Finally, for the definition of the most suitable number of groups present in the input data, the following rules were applied: (1) if both the longest and the last stable plateau resulted in a cluster number higher than the default APC, use the default; (2) else, if only the longest plateau is lower than the default, use the longest plateau value; and (3) if both the longest and the last stable plateau are lower than the default, use the last stable plateau to set the number of groups. This number of groups was then used to calculate the grouping of the input dataset using the function for AHC from the APC package. The described grouping was applied for the desired number of subgrouping levels (ranks below the species level). The R code used for these calculations is available as Supplementary Material.

In order to test the impact of the number of steps and the minimum number of iterations to use as the cut-off for definition of the last plateau for the determination of the group number, we used the described test datasets. We ran all calculations with all possible combinations of different step numbers (1,000; 2,000; 5,000; 10,000), minimum plateau lengths (sliding window size 1 per cent, 0.5 per cent, 0.25 per cent, 0.1 per cent, or 0.01 per cent of the step number), and minimum group members to have as input for further subgrouping (5, 7, and 10). In these tests, we found that the coherence of grouping by the described workflow and the phylogenetic trees increased with the number of steps and with the reduction of the sliding window size applied for plateau determination. Notably, with a fixed set of step number and sliding window size, the impact of the minimal group size increases with the increasing size of the input dataset. Since our initial tests showed that ambiguities in the sequences and, to a lesser extent, also duplicated sequences negatively impact the grouping by the described workflow, we also tested the different test datasets without duplicate sequences and sequences with ≥10 ambiguous nucleotides or gaps. Here, we present results from datasets without sequences having ≥10 ambiguous nucleotides or gaps and only retained one representative for sequences sharing 100 per cent nucleotide identity. Unless indicated, the used parameter set for the presented results was 10,000 steps, sliding window proportion resulting in sliding window length of three, and minimum group size of five.

#### 2.6.6 Proposal for WNV group designations

**Figure 1.**
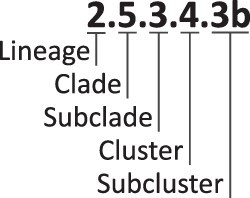
Graphical representation of the proposed hierarchy and the corresponding group labels. The levels of the proposed hierarchy are ordered top to bottom; the corresponding group label is organized left to right. Note that the subcluster can either have the number only or the number combined with a letter.

Alongside our new workflow, we here propose to use a generic nomenclature based on a hierarchical numbering system. This proposal is outlined in Fig. 1. Based on the use of designations in the literature, we propose to designate the levels within the species WNV descending from the species through lineage, clade, subclade, cluster, and finally subcluster. The subclusters can additionally be divided further, then carrying a letter as the suffix. The digits representing the different hierarchical levels are separated by a ‘.’ (compare Fig. 1). Here, we examined the grouping in different depths as indicated for the respective analyses. With the lineage designations, we followed the established lineage numbering; hence, where necessary, lineage designations automatically assigned in the calculations were replaced by the corresponding established designations.

#### 2.6.7 Combination of the clustering workflow, phylogenetic analyses, and geolocation

The assigned hierarchical levels of WNV sequences detected in Germany from 2018 to 2020 were summarized per new phylogenetic group, collection year, and sample type (wild/captive bird, horse, mosquitoes, and humans). These were exported as a CSV file into the QGIS Desktop (v3.16.15).

## 3. Results and Discussion

Originally, the goal of this study was to provide an update on the WNV epizootic in Germany in 2020. However, we encountered significant problems in consistently allocating WNV sequences into different groups below the species rank, namely:

1. the lack of objective grouping due to undefined demarcation criteria for the splitting of sequences into groups, resulting in arbitrarily adjusted groupings, and
2. the missing common group designations below species level within the *West Nile Virus* species (and in general) that together with the used nomenclature, which often relies on geographical terms that, due to the spread of the virus no longer fit, result in misleading designations.

### 3.1 Proposal for a hierarchical WNV nomenclature below the species level

To date, there is no commonly used system in the WNV research community for the definition and designation of virus groups below the species level. Rather, a substantial number of ways to define and of terms to designate virus groups at different levels of a hierarchical system below the species are used. These are also different from what is used for other virus species and what is commonly understood (see Table 2).

**Table 2.** Overview of terms commonly used for the designation of virus sequences into groups below the species rank.

		Current common use in the WNV research community			Proposed use	
Term	General definition	Definition	Example Lineage 1	Example Lineage 2	Level below species/term	Example designation
Lineage	Rank-independent term for the relationships between ancestors and descendants through time; ‘a lineage […] is a diachronic concept, a series of ancestors and descendants’ (Cellinese, Baum, and Mishler 2012; Mishler 2010)	Broadest monophyletic group below the WNV species rank. There are nine proposed WNV lineages (Fall et al. 2017)	Lineage 1; Lineage 1a	Lineage 2	1/Lineage	Lineage 1; Lineage 2
Clade	Rank-independent term for a monophyletic group on a phylogenetic tree; a clade is a synchronic monophyletic group that comprises ‘all and only descendants of a common ancestor’ (Cellinese, Baum, and Mishler 2012; Mishler 2010)	Smaller monophyletic group within the lineage. Typically denoted with letters. Example, 1a–1c and 2a–2d (May et al. 2011; McMullen et al. 2013). In Lineage 2, this level also describes a monophyletic group sharing similar geographic range (Ziegler et al. 2020)	Clade 1a	Clade 2d; Central/Southern European clade	2/Clade	Clade 1.1; Clade 2.5
Subclade	‘A smaller monophyletic group contained within a larger clade’ (Campbell et al. 2022)	Smaller monophyletic group within the clade. More often used in Lineage 2. In Lineage 2, these are also used to describe sequences from a monophyletic branch sharing geographic range (Barzon et al. 2015; Ziegler et al. 2020)	Not commonly used in Lineage 1	Central/Southern European subclade; subclade: ‘Eastern German clade’	3/Subclade	Subclade 1.1.4; Subclade 2.5.1
Cluster	A cluster is a discrete category into which biological diversity typically falls (Hassan et al. 2017); in regard to sequences a cluster is ‘a diverse set of sequences […] with high similarity’ (Balaban et al. 2019)	Smaller monophyletic group within the clade in Lineage 1, sharing a single ancestor and or a fixed unique non-synonymous mutation (May et al. 2011). Smaller monophyletic group within the clade or subclade in Lineage 2 (Barzon et al. 2015)	Cluster 1	Italian Lombardy cluster	4/Cluster	Cluster 1.1.4.1; Cluster 2.5.1.1
Subtype	A subset of a genotype based on a certain characteristic (Stadejek et al. 2006; Stadejek et al. 2008)	A group of sequences, which share fixed non-synonymous mutation(s) and/or sometimes common geographic origin (May et al. 2011) (Mann et al. 2013) (occasionally used interchangeably with genotype)	Mediterranean subtype (cluster 2)	Not commonly used in Lineage 2	5/Subcluster (designated by fifth decimal place)	Subcluster 1.1.4.1.6; Subcluster 2.5.1.1.5
Genotype	Genotype ‘describes an organisms complete set of genes’ (Nature Education 2023); in viral phylogenetics also understood as ‘monophyletic cluster of sequences with high statistical support’ (Goya et al. 2020)	A group of sequences, which share fixed non-synonymous mutation(s) and/or sometimes common geographic origin (May et al. 2011) (Mann et al. 2013) (occasionally used interchangeably with subtype)	NY99 genotype (cluster 4)	Not commonly used in Lineage 2	6/Subcluster (designated by a letter as suffix)	Subcluster 1.1.4.1.6a; Subcluster 2.5.1.1.5a

**Figure 2.**
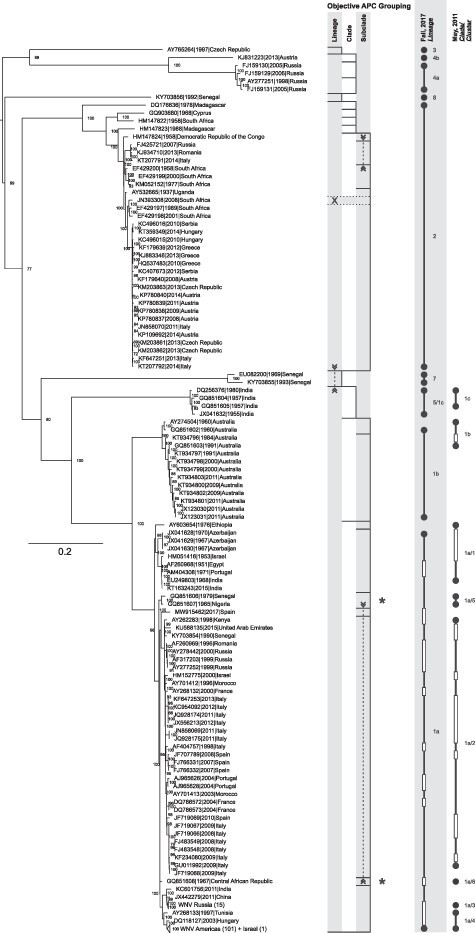
Comparison of the group structures of APC groupings of Test Dataset TD03 with previously defined groupings and phylogenetic reconstruction. The representation of the objective APC grouping includes the addressed hierarchical levels, starting with lineage, decreasing from left to right down to the subclade. The vertical lines mark the final level down to which the grouping could be done (limited either by the minimum group size applied for the input of subgrouping or by the hierarchical level that was the last to be shown). Horizontal lines separate the individual groups. Dashed vertical lines with arrows connect areas of the graph together forming one common group interspersed by other group(s), an example is marked ‘*’. The horizontal rectangle with dashed lines and labeled ‘X’ marks a sequence that was not considered for APC/AHC grouping due to its high number of ambiguities (≥10). For comparison, the groupings that were previously published by Fall et al. (2017) and May et al. (2011) are included. Here, a filled circle represents a singleton sequence making up the respective group as labeled and two filled circles connected by a vertical line represent a larger group. Unfilled rectangles mark sequences included in the tree but not part of the cited analyses. The ML phylogenetic analysis of sequences from TD03 was done with the best fitting model GTR + I + G and 100,000 ultrafast bootstraps. Few large branches consisting of sequences from almost the same geographic regions are collapsed into triangles. The nodes are labeled with ultrafast bootstrap values.

The designations of the hierarchical levels *inter alia* include the terms ‘lineage’, ‘clade’, and ‘cluster’ (Fig. 2). However, the use of the labels to designate different levels of the hierarchical system is variable. The WNV research community especially uses the term ‘lineage’ to describe a broader hierarchical group consisting of clades and/or subclades, while in other virus species, such as severe acute respiratory syndrome coronavirus 2 and rabies virus (RABV), the term ‘clade’ defines a broader monophyletic group consisting of subclades and lineages (Rambaut et al. 2020; Campbell et al. 2022).

Moreover, beside the variable use of terms for the designation of hierarchical levels, the criteria used to define the groups are variable. For instance, Aguilera-Sepulveda et al. (2021), Barzon et al. (2015), and May et al. (2011) defined clusters found within WNV Clade 1a as sequences belonging to a monophyletic group with a close phylogenetic relationship, with a common ancestor and fixed and unique aa substitutions. In another example, McMullen et al. (2013) defined four clades (Clades 2a–2d) based on nucleotide identities and monophyletic branching within the phylogenetic tree. However, the demarcation criteria regarding nucleotide identities or aa similarities for each clade were not clearly defined.

**Figure 3.**
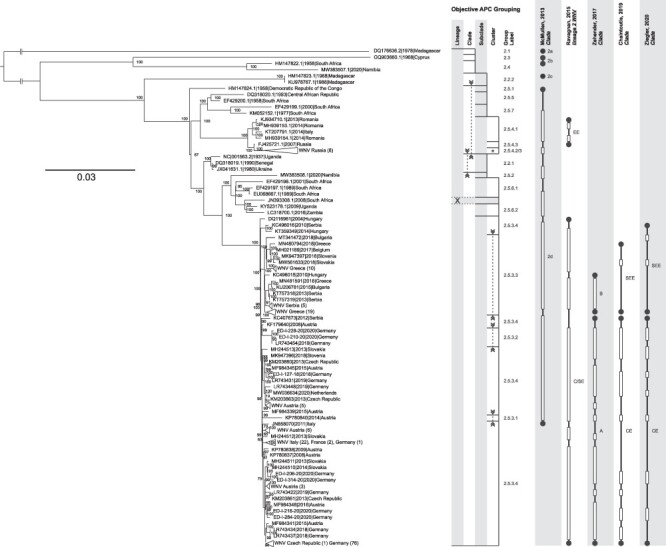
Comparison of APC groupings of WL2 sequences with previously defined groupings and phylogenetic reconstruction. The representation of the objective APC grouping includes the addressed hierarchical levels, starting with lineage (not calculated here but WL2 sequences included according to published references), decreasing from left to right down to the cluster. The vertical lines mark the final level down to which the grouping could be done (limited either by the minimum group size applied for the input of subgrouping or by the hierarchical level that was the last to be shown). Horizontal lines separate the individual groups. Each group is labeled at the right-hand side of the graph. Dashed vertical lines with arrows connect areas of the graph together forming one common group interspersed by other group(s). The horizontal rectangle with dashed lines and labeled ‘X’ marks a sequence that was not considered for APC/AHC grouping due to its high number of ambiguities (≥10). For comparison, the groupings that were previously published by Chaintoutis et al. (2019), McMullen et al. (2013), Ravagnan et al. (2015), Zehender et al. (2017), and Ziegler et al. (2020) are included. Here, a filled circle represents a singleton sequence making up the respective group as labeled and two filled circles connected by a vertical line represent a larger group. Unfilled rectangles mark sequences included in the tree but not part of the cited analyses. The ML phylogenetic analysis of sequences from WL2 was done with the best fitting model GTR + I + G and 100,000 ultrafast bootstraps. Few large branches consisting of sequences from almost the same geographic regions are collapsed into triangles. The nodes are labeled with ultrafast bootstrap values.

Likewise, the labels used to designate the groups are diverse. Often, groups are labeled according to their first geographic occurrence. Although geographic labels may provide epidemiological information regarding the origin of the WNV cases, these descriptive labels can cause misrepresentation. For instance, the geographic range of WNV cases designated to the Lombardy cluster, which consisted of WNV cases from Lombardy, Italy, as of 2015 (Barzon et al. 2015), is recently expanding. The Lombardy cluster now also includes WNV sequences from France and Spain (Aguilera-Sepulveda et al. 2022). Similarly, WNV Clade 2d sequences from the European continent were designated according to the supposed region of the viruses origins, like WNV sequences from Russia and Romania that were designated as the Eastern European Lineage 2 WNV (labeled EE in Fig. 3) (Ravagnan et al. 2015; Cotar et al. 2018) or WNV sequences from Hungary, Austria, Greece, Serbia, and Italy that were put into the Central/Southern European Lineage 2 WNV (labeled C/SE in Fig. 3) (Chaintoutis et al. 2019; Ziegler et al. 2020).

Due to the issues outlined earlier, we set out to design a novel unified system for the hierarchical organization of WNV (and other viruses) based on (1) an objective definition of subgroups (see Section 2.6.5 and Section 3.2), (2) a defined set of names for the different nested hierarchical levels, and (3) a system for group designations that does not rely on geographic or other names that can likely be subject to change. Although we acknowledge the importance of a universal designation below the species rank encompassing all virus species, we in part still followed the conventional designation of WNV sequences below the species rank to prevent any confusion. For the species *West Nile virus*, we define a term associated with a specific hierarchical level, as summarized in Table 2. We propose to use the following order of hierarchical groups based on increasing shared genetic identities within the group: lineages (highest level below the species, as commonly used in the WNV community, Level 1), clade (Level 2), subclade (Level 3), cluster (Level 4), and subcluster (Level ≥ 5). Moreover, we propose to utilize a generic nomenclature for the defined groups based on a hierarchical numbering system to designate each group at different hierarchical ranks in a logical and standard manner (‘Proposed use’ in Table 2). These generic labels also provide information regarding the hierarchical level through the number of decimal and/or alphabetical places included (compare Fig. 1). Furthermore, these generic labels can be used continuously even when the group members do not share particular characteristics, such as geographic origin. Finally, we applied these proposals to WNV sequences from previously published studies and members of WL2 available in the public database to compare our results with previous classifications.

### 3.2 Application of the developed grouping workflow yields reasonable groups

To address the aforementioned grouping issues, we developed a workflow for objective clustering of sequences into different hierarchical groups below species level. This clustering workflow employs APC, which is a non-hierarchical mathematical clustering method, with AHC to split the dataset into groups. This workflow is based on the works of Fischer et al. (2018), who initially utilized APC to define objective clusters of RABV sequences. Their group also developed the plateau method to determine the number of clusters in a given dataset, typically a user-defined parameter required in clustering programs such as hierBAPS (Cheng et al. 2013), Cluster Picker (Ragonnet-Cronin et al. 2013), TreeCluster (Balaban et al. 2019), and PhyCLIP (Han et al. 2019). Furthermore, the workflow of Fischer and colleagues only requires pairwise identities between all pairs of virus sequences as input. Overall, the method overcomes the need for inputting subjective criteria such as number of clusters, the minimum number of sequences per cluster, or support thresholds for cluster allocation. While Fischer and colleagues successfully assigned RABV and *Francisella tularensis* isolates into reasonable objectively defined clusters (Fischer et al. 2018; Busch et al. 2020), the APC results were partly incongruent with the branching of a RABV phylogenetic tree. This incongruence is potentially caused by the non-hierarchical clustering properties of the APC algorithm in contrast to the phylogenetic analysis (Fischer et al. 2018) but could also be caused by an uncertainty of the suitable number of clusters present in the dataset. Therefore, to improve the workflow, we further developed the determination of the number of clusters and included AHC to determine the generated clusters. In order to define multiple hierarchical levels, the method was iteratively applied to subsets of the data corresponding to the subgroups of the preceding iteration, i.e. higher level in the hierarchy. For optimization of the parameters, we repetitively analyzed the described test datasets and compared the results with the grouping as described in the respective studies (McMullen et al. 2013; Ravagnan et al. 2015; Zehender et al. 2017; Chaintoutis et al. 2019; Ziegler et al. 2020). We found that the minimum number of sequences per group to be used as input for further subgrouping and the number of iterations used to define the plateau (the window size) had the major impact on the results. On the contrary, the overall number of iterations applied to determine the number of clusters only had less influence. The optimal parameters used for all subsequent analyses were the window size of three for the determination of the longest and last stable plateaus, respectively, and the group size of five that was necessary to further split the group. In order to ensure that the number of iterations did not limit the quality of the clustering, we used 10,000 iterations throughout.

We initially applied the developed workflow with the settings outlined in the previous paragraph to the test dataset TD03 for the definition of groups within the three proposed levels: ‘lineage’, ‘clade’, and ‘subclade’. Figure 2 shows the group structure obtained for TD03. According to the used minimum size of a group to be used as an input for further subdivision in the next lower hierarchical level, the grouping stopped at different levels of the hierarchy. Overall, the objective APC grouping coincides with groups that would be defined when analyzing the tree visually. Most groups we found fit with the traditional definition of a phylogenetic group being monophyletic. In case of the grouping result for TD03, however, we received split groups (in the graph connected with a dashed line with arrows pointing inward) that were not intuitively clear at the first glance at the tree. For instance, the subclade combining the Clusters 1a/5 and 1a/6 of May and colleagues (2011) (marked ‘*’ in Fig. 2) was unexpected because it is not monophyletic. This subclade is split into two parts (interspersed by two other subclades). This split is possible since our workflow mainly depends on the nucleotide identities of pairwise aligned sequences but not on reconstructed hierarchical connections. Looking at the tree in more detail, it becomes clear that unexpected groupings appear where the observed ultrafast bootstrap values are low (according to the IQ-Tree documentation, only values above 95 per cent indicate trustworthy groups (Minh et al. 2022)) and/or the branch lengths between the subclade members are indeed quite short and therefore the observed groupings make sense. Hence, we proceeded with the proof of concept for the developed method.

### 3.3 Proof of concept for the developed clustering workflow

For the proof of concept, we were first interested in the grouping structure and compared our grouping results with published groupings. Using the abovementioned parameters, we could reproduce the groupings of the individual Test Datasets TD01 and TD02 as published (May et al. 2011; Fall et al. 2017) (results not shown). For the Combined Dataset TD03, we obtained the group structure as shown in Fig. 2. Both Rizzoli et al. (2015) and Fall et al. (2017) categorized WNV Lineages 1a, 1b, 1c, 4a, and 4b (4/9) as distinct and separate lineages, while May et al. (2011) designated the same groups of sequences as Clades 1a, 1b, and 1c which they further subdivided into clusters. As can be seen, the objective grouping of the APC/AHC workflow ultimately coincides with the previously performed groupings, albeit at different levels of the hierarchy and hence with different labels. Although the APC/AHC workflow at the first level (lineage) groups the previously defined (Fall et al. 2017) Lineages 1a, 1b, 1c, and 2 and 4a and 4b, respectively, at the next level (clade), our workflow divides the fused lineages into individual clades corresponding with the previously defined Lineage 4b (Fall et al. 2017), while Lineage 4a (Fall et al. 2017) was subdivided into two clades. Likewise, Lineages 1a, 1b, 1c, and 2 (Fall et al. 2017) correspond to individual clades. At the subclade level, the clusters that May et al. (2011) defined within Lineage 1a match our subclades quite well: the members of Cluster 1a/2 (May et al. 2011) are comprised within a single subclade; sequences of Cluster 1a/1 are divided into two subclades; Clusters 1a/3 and 1a/4 are fused at the subclade level but are subdivided at the cluster level (not shown); finally, Clusters 1a/5 and 1a/6 are also combined into one subclade, which notably is not a monophyletic group, but all its branches in the phylogenetic tree descend from the same branch and their branch lengths are very short. Therefore, the co-allocation by APC/AHC is congruent with the minor distances that are visible in the phylogenetic tree. In summary, although in detail there are differences, overall, the developed objective grouping by APC/AHC yields meaningful and reliable groupings.

**Figure 4.**
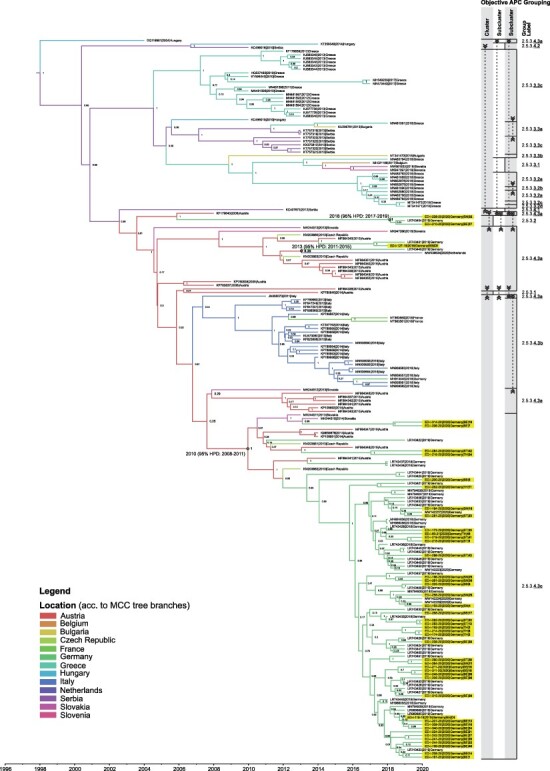
Bayesian MCC tree representing time-scaled phylogeny of European WNV Subclade 2.5.3 complete coding sequences together with objective APC groups. WNV sequences acquired in this study are highlighted with color. All other WNV sequences were retrieved from GenBank and are listed in Table S3. The colored branches of MCC trees represent the most probable geographic location of their descendants (see legend ‘locations’). Bayesian pp are indicated at each node. Time (in years) is indicated as x-axis below the MCC tree. The time for the MRCA, time intervals defined by the 95 per cent HPD, and pp are shown in the following nodes that consist of the following WNV sequences: (1) LR743448 and MW036634, (2) Cluster 2.5.3.2 sequences, and (3) Subcluster 2.5.3.4.3c sequences. The representation of the objective APC grouping includes the addressed hierarchical levels, starting with cluster decreasing from left to right down to the subcluster. The vertical lines mark the final level down to which the grouping could be done (limited either by the minimum group size applied for the input of subgrouping or by the hierarchical level that was the last to be shown). Horizontal lines separate the individual groups. Each group is labeled at the right-hand side of the graph. Dashed vertical lines with arrows connect areas of the graph together forming one common group interspersed by other group(s).

In addition to the abovementioned proof of concept for the separation of WNV of all lineages into the different hierarchical levels (lineages, clades, and subclades), we analyzed the WL2 complete coding sequences available in the International Nucleotide Sequence Database Collaboration databases and assigned them group labels according to the above-outlined rules. As stated for the first analysis, the grouping we received overall fits well with what is seen intuitively in the tree. Usually, the observed polyphyletic interspersed groups, e.g. Clades 2.2 and 2.5 in Fig. 3, which are in part associated with low ultrafast bootstrap values in the tree (according to the IQ-Tree documentation, only values above 95 per cent indicate trustworthy clades (Minh et al. 2022)) are resolved at the next lower grouping level (in this example at the subclade level). Here, Clade 2.2 (Fig. 3) is a polyphyletic group comprising five sequences, which are at the subclade level separated into Subclades 2.2.1 and 2.2.2. This interspersed grouping at the clade level, which occurs in the APC step based on the pairwise identities, cannot be resolved using AHC. This incongruency is due to the inherent non-hierarchical characteristics of the APC, as described by Fischer et al. (2018). Similarly, in the deeper grouping of Subclade 2.5.3 sequences, Subcluster 2.5.3.4.3a includes WNV sequences that are interspersed in the ML and MCC trees (Fig. 4). This subcluster formed a paraphyletic group in both ML and MCC trees and demonstrated low ultrafast bootstrap values (<80 per cent) and posterior probability (pp) values (<0.6), respectively.

The discussed topology in phylogenetic trees depicts the so-called ‘supercluster’, wherein divergent subgroups are nested within a more extensive cluster structure (Han et al. 2019). Therefore, in combination with phylogenetic trees, our grouping workflow can also provide insights regarding the source–sink ecological dynamics of WL2 in Europe. This dynamic has been previously discussed in the phylogeographic and phylodynamic analyses of Zehender et al. (2017) and Ziegler et al. (2020). Specifically, Cluster 2.5.3.4 may represent the putative source of the WNV population that gives rise to its subgroups, reflecting the trajectory and divergence of variants (Han et al. 2019). In parallel, members of Cluster 2.5.3.4 were detected in locations described as ‘radiation centers or sources’ of WL2 migration in Europe (e.g. Hungary and Austria). Furthermore, members of other WNV clusters were detected in areas described as ‘receiving areas or sinks’ of WNV migration, such as Greece (Cluster 2.5.3.3).

To further verify the workflow, we compared our grouping with previously published results of McMullen et al. (2013), Ravagnan et al. (2015), Zehender et al. (2017), Chaintoutis et al. (2019), and Ziegler et al. (2020). Noteworthy, all studies that were available for comparison only included partial sets of the sequences that we included here. The comparison of the results of the objective APC/AHC grouping and the clades defined by McMullen (McMullen et al. 2013) shows that there are two main differences between both: (1) McMullen’s Clade 2b is disrupted into Clades 2.3 and 2.4 in our grouping; this is likely caused by the inclusion of the 2020 sequence from Namibia (MW383507), which forms Clade 2.4 together with the 1958 South African sequence (HM147822) that was included in McMullen’s Clade 2b; (2) the sequences comprised in McMullen’s Clade 2d were now put into Clade 2.5, except for 1990 Senegal (DQ318019) and 1937 Uganda (NC_001563) that form Clade 2.2 together with one 1988 sequence from Madagascar (HM147823). These two deviations show the expectable effect of addition of sequences on tree topology and sequence grouping. The comparison between the groupings of Ravagnan et al. (2015) and ours shows that the virus group designated ‘Eastern European Lineage 2 WNV’ (labeled EE in Fig. 3) coincides with our Subclade 2.5.4 and those of the ‘Central/Southern European Lineage 2 WNV’ (labeled C/SE in Fig. 3) are all grouped into Subclade 2.5.3. In the studies of Zehender et al. (2017), Chaintoutis et al. (2019), and Ziegler et al. (2020), viruses belonging to Ravagnan’s C/SE Lineage 2 WNV (Ravagnan et al. 2015) were subdivided into two groups. These were labeled Clade A (Zehender et al. 2017) or Central and Eastern European clade (CEC; Chaintoutis et al. 2019; Ziegler et al. 2020) and Clade B (Zehender et al. 2017) or Southeastern European clade (SEEC; Chaintoutis et al. 2019; Ziegler et al. 2020), respectively. Using our APC/AHC workflow, they are grouped together in Subclade 2.5.3. At the next hierarchical level (cluster), with a single exception (LR743454, Germany 2019, Cluster 2.5.3.2), Clade A/CEC is completely comprised within Cluster 2.5.3.4. Likewise, Clade B/SEEC is fully comprised in Cluster 2.5.3.3, except for the two sequences from Hungary 2014 (KT359349) and Serbia 2010 (KC496016). Interestingly, Cluster 2.5.3.1 comprises only a single WNV sequence from Austria (KP780840) that has not been included in previous phylogenetic studies (Chaintoutis et al. 2019; Ziegler et al. 2020) since it was considered an outlier based on its temporal signal relative to other WNV Subclade 2.5.3 sequences. This sequence also showed the lowest pairwise nucleotide identities among members of Subclade 2.5.3. Noteworthy, Ziegler and colleagues highlighted that LR743454 formed its own distinct subclade within the CEC. In our analysis, this sequence received two companions, altogether forming Cluster 2.5.3.2.

Since the introduced workflow is designed to enable the objective grouping of sequences also in dynamic situations, i.e. when frequently new sequences are available as it is the case in Germany in recent years, we assessed the impact of sequence additions by a jackknife experiment with 1,000 repeats. For this experiment, we used the WL2 dataset from which we randomly removed between 1 and 15 sequences before calculating the groupings of the partial datasets. Thereafter, we compared the grouping in the smaller dataset, in this experiment representing the earlier point in time, with the grouping in the complete dataset, i.e. the later dataset. To determine the effect, we checked how many of all pairs of sequences were concurrently grouped, i.e. were found in the same or in different groups before and after the addition of sequences. In this analysis, in 31.7 per cent of the performed pairwise analyses, more than 99 per cent of all relations remained unchanged, irrespective of the number of removed sequences (minimum 1, first quartile 3, median 6, third quartile 10, maximum 15). In 2.8 per cent of the analyses, the removal/addition of up to six sequences did not impact the grouping at all, and all relations remained identical. Moreover, we found that individual sequences apparently could eventually have a higher impact than the mere number of sequences removed/added. For instance, Sequence MW383507.1, which above we speculate leads to changes in the grouping in comparison with published results, was among those that were frequently found in analyses with strong deviations (less than 80 per cent concurrent grouping) but never in analyses with identical grouping. Of course, removal/addition of other sequences may balance the effects; therefore, this is no absolute correlation. In summary, there are both qualitative and quantitative effects that affect the groupings. Hence, a dynamic situation with frequent additions of new sequences will lead to changes in the grouping and consequently in the group designations if nothing is done to prevent this. Although changes in the designations reflect changes in the situation, care must be taken to ensure that no conflicting labels will be issued. This, of course, has to be solved at the programming level.

Taken together, the presented comparisons between published studies and the grouping obtained by application of the newly developed APC/AHC workflow show that our objective workflow reliably puts sequences into meaningful groups.

### 3.4 WNV circulation in Germany extended in space and species

**Figure 5.**
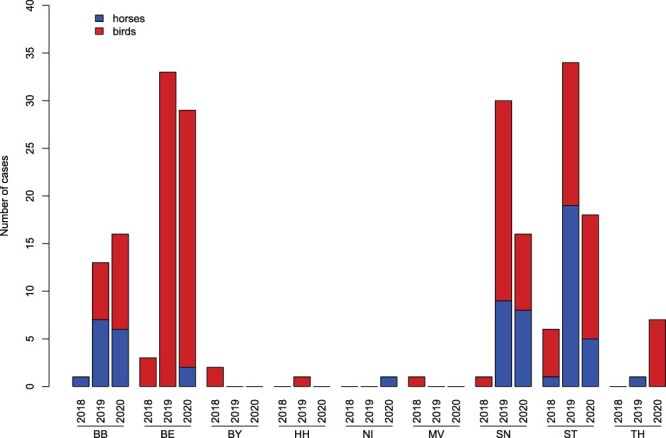
Notifiable WNV cases of birds and horses in Germany from 2018 to 2020. The number of cases was summed up per federal state and year. Notifiable cases in horses and birds were represented by blue and red bars, respectively. Abbreviations for federal states in Germany: BB, Brandenburg; BE, Berlin; BY, Bavaria; HH, Hamburg; NI, Lower Saxony; MV, Mecklenburg-Western Pomerania; SN, Saxony; ST, Saxony-Anhalt; and TH, Thuringia.

In 2020, we detected 65 birds (captive = 33 and wild = 32) and 22 horses that tested positive for WNV in Germany (diagnosed between 14 July and 20 October and two retrospective cases from 2021). All but one WNV-positive bird succumbed to infection (Table 1; no. 44). The number of notifiable cases of WNV in birds and horses in 2020 is similar to the previous year, particularly in regions with the highest WNV activity, i.e. Berlin, Saxony, and Saxony-Anhalt (Fig. 5; Ziegler et al. 2020). However, we observed an increasing number of WNV cases in Brandenburg, Thuringia, and Lower Saxony. All WNV-positive birds and horses detected in 2020 were found in federal states which also reported WNV cases in 2018 and 2019 (Fig. 5 and S1) except for a new WNV case detected in Lower Saxony. Notably, all twenty-two probable autochthonous human WNV cases in 2020 occurred in these federal states (Berlin = 7; Saxony = 11; Saxony-Anhalt = 4) (European Centre for Disease Prevention and Control 2020; Pietsch et al. 2020; Frank et al. 2022). Therefore, this kind of WNV surveillance in both wildlife and captive animals could provide an early warning for autochthonous WNV infection in humans in Germany. Hence, reports of WNV infection in birds and horses in an area must be provided promptly (e.g. updates of FLI websites) to advise the medical community and the public, regarding a potential risk of WNV infection in specific regions in Germany, as well as the risks in blood transfusion and organ transplantation safety. Although vaccines against WNV disease in humans are still under development (Ulbert 2019), clinicians must be aware of the potential presence of WNV circulation in the local region to reach a correct diagnosis since WNV diagnostics is not routinely performed in Germany (Schneider et al. 2021).

Here, we report the first case of a WNV infection in Lower Saxony, where a horse with WNV-specific IgM antibodies was detected in the district of Helmstedt, and the first case of WNV-infected birds in Thuringia, particularly in the districts of Erfurt and Gera (Figs. 5 and S1). We also reported the first cases of WNV infection in three districts in Brandenburg (i.e. Teltow-Fläming, Barnim, and Dahme-Spreewald) and in one district in Saxony-Anhalt (Börde) (Fig. S1). Areas with reported WNV infection match with areas with high average temperatures (>20°C), lower average precipitation (≤250 mm), and lower average climatic water balance (−150–50 mm) in summer 2020 (Fig. S2) (Deutscher Wetterdienst 2020). Higher average temperatures over several days may increase the risks of WNV transmission through mosquito vectors (Holicki et al. 2020). The higher average temperatures in these areas probably caused the epizootic emergence of WNV by shortening the extrinsic incubation period in local mosquito populations. Furthermore, the WNV activity is more likely to increase during drought than during rainy periods (Paull et al. 2017). It is also possible that the declining water sources force the avian reservoir hosts to aggregate, increasing the probability of contact between birds and mosquitoes and WNV transmission (Paull et al. 2017). However, we did not detect the re-emergence of WNV in Hamburg and in two districts in Brandenburg (Ostprignitz-Ruppin and Havelland) in 2020, despite the observed higher average temperatures (>20°C) and lower average precipitations (126–200 mm) in summer 2020.

We also detected WNV infections in twenty-one different bird species from six taxonomic orders (Table 3). The majority of WNV-infected avian species are classified as birds of prey (order *Accipitriformes*, 29 per cent), followed by songbirds (order *Passeriformes*, 26 per cent), captive flamingos (order *Phoenicopteriformes*, 23 per cent), and owls (order *Strigiformes*, 17 per cent). Most of the WNV-infected bird species in 2020 were also reported in an earlier study (Ziegler et al. 2020), except for the alpine chough (*Pyrrhocorax graculus*), Bohemian waxwing (*Bombycilla garrulus*), and golden eagle (*Aquila chrysaetos*). However, all three species belong to taxonomic orders that were already described before to be repeatedly affected by WNV (*Passeriformes* and *Accipitriformes*) (Michel et al. 2018, 2019). Notably, the golden eagle from Brandenburg (no. 44) is the only reported case in 2020 that recovered from WNV infection (Table 1).

**Table 3.** Summary of avian species infected with WNV in 2020 in Germany.

Order	Common English name	Scientific name	Housing	Number	Affected federal state^a^
*Accipitriformes*	Unspecified buzzard	*Buteo* sp.	Wild	1	SN
	Northern goshawk	*Accipiter gentilis*	Wild/captive	17	BE, BB, SN
	Golden eagle	*Aquila chrysaetos*	Captive	1	BB
*Charadriiformes*	Black-tailed gull	*Larus crassirostris*	Captive	1	BE
*Passeriformes*	Alpine chough	*Pyrrhocorax graculus*	Captive	1	ST
	Blue tit	*Parus caeruleus*	Wild	8	BE, SN, TH
	Eurasian jay	*Garrulus glandarius*	Wild	1	TH
	European greenfinch	*Carduelis chloris*	Wild	1	BB
	Domestic canary	*Serinus canaria forma domestica*	Captive	1	ST
	Great tit	*Parus major*	Wild	1	SN
	Hooded crow	*Corvus corone cornix*	Wild	2	BE
	Bohemian waxwing	*Bombycilla garrulus*	Captive	1	BB
	Unspecified sparrow	*Passer* sp.	Wild	1	SN
*Phoenicopteriformes*	Chilean flamingo	*Phoenicopterus chilensis*	Captive	6	BE, ST, SN
	American flamingo	*Phoenicopterus ruber*	Captive	1	BE
	Unspecified flamingo	*Phoenicopterus* sp.	Captive	8	BE, ST, TH
*Psittaciformes*	Swift parrot	*Lathamus discolor*	Captive	2	ST
*Strigiformes*	Snowy owl	*Bubo scandiacus*	Captive	4	ST, TH
	Little owl	*Athene noctua*	Captive	5	BB
	Barn owl	*Tyto alba*	Wild	1	BB
	Eurasian eagle-owl	*Bubo bubo*	Captive	1	ST

a Abbreviations for federal states: BE, Berlin; BB, Brandenburg; SN, Saxony; ST, Saxony-Anhalt; TH, Thuringia.

### 3.5 Update on the WNV situation in Germany, 2020

After we had validated the workflow, we analyzed the ongoing WNV epizootic in Germany using this tool. The result of grouping sequences that belong to Subclade 2.5.3, to which all viruses circulating in Germany until 2020 belong, is shown in Fig. 4. As can be seen, Subclade 2.5.3 can be further subdivided into the four Clusters 2.5.3.1, 2.5.3.2, 2.5.3.3, and 2.5.3.4. Interestingly, Cluster 2.5.3.1 only comprises the aforementioned Austrian sequence KP780840 that was previously deemed an outlier and therefore disregarded in previous analyses (Chaintoutis et al. 2019; Ziegler et al. 2020). Cluster 2.5.3.2, which can, due to the group size restriction, also not be further subdivided, consists of three German sequences (LR743454 from 2019 and no. 32 and no. 37 from 2020), also mentioned earlier. On the contrary, Clusters 2.5.3.3 and 2.5.3.4 can be further subdivided into multiple subclusters each. Although, to a large extent, the detected subclusters comprise sequences from individual countries, they are clearly not geographically homogenous, highlighting the problem of geographic criteria for the designation of phylogenetic groups. For instance, Subcluster 2.5.3.4.3b mainly comprises not only sequences from Italy but also two sequences from France and one sequence of a case imported to Germany (MH910045). Likewise, Subcluster 2.5.3.4.3c, into which the majority of WNV sequences from Germany were grouped, also comprises sequences from Slovakia (*n* = 2), Austria (*n* = 5), and the Czech Republic (*n* = 2).

**Figure 6.**
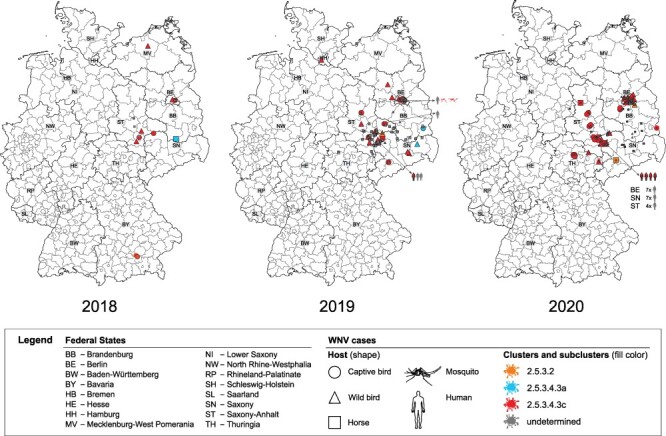
Geographic distribution of WNV cases in Germany from 2018 to 2020 per host and (sub)cluster. Labeling according to the legend in the graph. WNV-positive cases confirmed by the national reference laboratory without complete coding sequences are labeled ‘undetermined’ in the legend.

**Figure 7.**
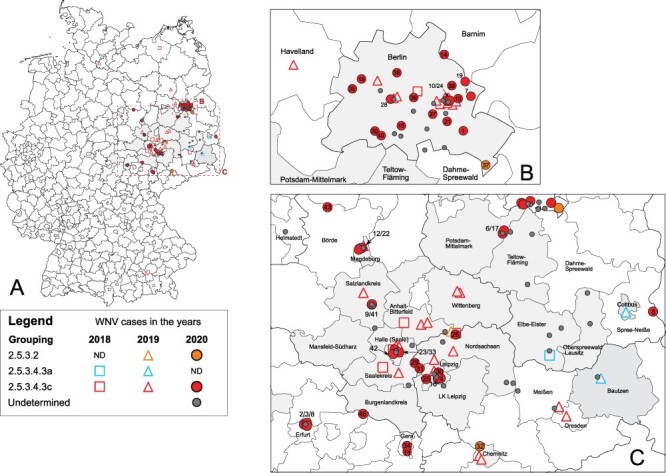
Summarized geographic distribution of WNV cases in Germany from 2018 to 2020. Labeling according to the legend in the graph. WNV-positive cases confirmed by the national reference laboratory without complete coding sequences are depicted in gray (labeled ‘undetermined’ in the legend). Districts depicted shaded in the maps indicate areas with (additional) WNV-positive cases from WNV seasons 2018–9 without a complete coding sequence. (A) Overview of the geographic distribution of WNV cases in Germany. Areas with high WNV activity in 2020 are shown in enlarged and separated maps, (B) Berlin, (C) Saxony, Saxony-Anhalt and Thuringia. New WNV cases from this study are indicated with numbers as described in Table 1

As summarized in Figs 6 and 7, sequences from WNV circulating in Germany from 2018 to 2020 were allocated to Cluster 2.5.3.2 and Subclusters 2.5.3.4.3a and 2.5.3.4.3c, respectively. A sequence of Cluster 2.5.3.2 was first detected in 2019 (LR743454) and previously formed an outlier (Ziegler et al. 2020), but now two additional viruses of this cluster were detected (ED-I-228-20—no. 32, ED-I-210-20—no. 37) (Fig. 6). The MRCA of WNV in Cluster 2.5.3.2 (see Fig. 4) was estimated to have existed around 2018 (95 per cent highest posterior density or 95 per cent HPD: 2017–19; Bayesian pp: 100 per cent). Unlike viruses of Cluster 2.5.3.2, viruses of Subcluster 2.5.3.4.3a were only detected in 2018 (ED-I-127-18—C5) and 2019 (LR743431 and LR743448), but not in 2020 (Fig. 6). The reasons for these rare detections remain elusive. Given the available information, we cannot determine whether Cluster 2.5.3.2 and Subcluster 2.5.3.4.3a have no established populations in Germany and hence must have been sporadically introduced to Germany in separate events. We may also have missed these clusters and subclusters as we could not sequence all WNV-positive cases from 2018 to 2020, e.g. no. 45 in Table 1. In some cases, simply, the sample quality and/or quantity prevent from generating the genome sequences, despite the use of the WNV multiplex-PCR-based high-throughput sequencing approach (Sikkema et al. 2020). For instance, most horse samples are serologically WNV IgM positive but WNV RNA negative, preventing the successful sequencing of WNV genomes. Moreover, organ materials from small passerines were often depleted after necessary routine diagnostics at the regional veterinary laboratories for other relevant avian viruses or after confirmatory diagnostics at the national reference laboratory at the FLI. Besides these technical issues, failure to detect WNV may be caused by infections occurring without clinical signs being noticed. For instance, Ziegler et al. (2022) described seropositive findings especially for *Passeriformes* or *Columbiformes* in endemic regions of Germany with high infection pressure. Moreover, we cannot exclude a remaining risk to miss virus detection because deceased birds might not be found, especially in sparsely populated areas. In the future, the genomic surveillance should therefore be enhanced by the inclusion of additional samples from mosquito surveillance (Kampen et al. 2020; Kampen, Tews, and Werner 2021), which can now easier be sequenced using the newly established protocol of Sikkema et al. (2020). This might help determine virus prevalence, diversity, and geographical distribution more comprehensively.

Beside the abovementioned two minor groups, the vast majority of WNV circulating in Germany were allocated to Subcluster 2.5.3.4.3c, which comprises all sequences previously allocated to the EGC plus additional sequences, *inter alia* two previously defined minor subclades comprising sequences LR743422 and LR743437/LR743434 (Ziegler et al. 2020). The EGC was dominant among the WNV that circulated in Germany from 2018 to 2019 and was characterized by a unique non-synonymous mutation (Lys_2114_Arg) located within the NS3 encoding genome region (noteworthy, LR743444 and LR743425 were previously designated into the EGC but do not harbor this mutation). This mutation no longer is a marker of the respective group (Subcluster 2.5.3.4.3c) which also comprises sequences without that specific mutation. Overall, the grouping we now observed (one major subclade and two minor (sub)clusters) agrees with our previous WNV report, wherein we detected six distinct ‘subclades’ circulating in Germany in 2018 and 2019 (Ziegler et al. 2020).

We estimated that the MRCA of the monophyletic branch consisting of Subcluster 2.5.3.4.3c sequences existed around 2010 (95 per cent HPD: 2008–11; pp: 100 per cent). Despite the fact that the vast majority of the 2.5.3.4.3c sequences are from Germany, it appears highly unlikely that the ancestors of that subcluster evolved in Germany, because no WNV cases were detected in Germany before 2018 despite the extensive arbovirus monitoring performed in the country since 2011 (Michel et al. 2019; Ziegler et al. 2022). Rather, given that (1) the estimated MRCA of the EGC coincided with large reported outbreaks in Eastern and Southeastern Europe (Rudolf et al. 2014; Sedlak et al. 2014; Jungbauer et al. 2015; Kolodziejek et al. 2015; Vlckova et al. 2015; Aberle et al. 2018; Kolodziejek et al. 2018) and (2) WNV complete genomes are not available from neighboring countries, we cannot determine where this subcluster diverged. Therefore, we hypothesize that members of the EGC were more likely introduced to Germany from neighboring countries in separate events and in a later time than its estimated MRCA.

While we detected Subcluster 2.5.3.4.3c all over the WNV-affected regions in Germany from 2018 to 2020, making it the dominating subcluster, viruses of (Sub)Cluster 2.5.3.2 and 2.5.3.4.3a were both in time and space restricted and of minor impact for the ongoing epizootic (Figs 6 and 7). Like with the sporadic occurrence of the aforementioned two (sub)clusters, there are also regions within Germany where WNV occurrence is only sporadic (regardless of the virus phylogenetic group). Namely, we detected WNV-infected wild birds in Rostock, Mecklenburg-Western Pomerania, in 2018 (*n* = 1) and in Hamburg (*n* = 1) and Havelland, Brandenburg (*n* = 1), in 2019. However, in these areas in the succeeding years, WNV activity was not reported.

As in the preceding years, in 2020, except for two cases in which viruses of Cluster 2.5.3.2 were detected, all other viruses were grouped into Subcluster 2.5.3.4.3c and in the same cities and districts as before (Fig. 7). In addition, viruses of Subcluster 2.5.3.4.3c were detected in three districts in Thuringia. These observations suggest that viruses of Subcluster 2.5.3.4.3c successfully established in local avian and mosquito populations in the affected regions, namely in Berlin, Saxony (particularly within Leipzig and neighboring areas), and Saxony-Anhalt, which led to the endemic circulation of WNV in these areas in 2020. We also observed the continuous geographic expansion of WNV belonging to Subcluster 2.5.3.4.3c from 2018 to 2020; however, only time will tell whether members of this subcluster successfully overwinter and establish themselves in these newly affected areas. In 2021, however, WNV cases in birds and horses were predominantly reported in Berlin, with a few additional WNV cases reported in Saxony, Saxony-Anhalt, and Brandenburg (Friedrich-Loeffler-Institut 2021).

WNV sequences within Subcluster 2.5.3.4.3c from Germany were acquired from mosquito pools (*n* = 2), horses (*n* = 2), and different bird species (*n* = 78) belonging to seven taxonomic orders. Complete coding sequences from five human WNV cases reported from 2019 (*n *= 1) to 2020 (*n* = 4) were also allocated into WNV Subcluster 2.5.3.4.3c (Fig. 6). We excluded a few human WNV cases where either only a partial genome sequence (*n* = 2) (Pietsch et al. 2020; Ziegler et al. 2020) or no sequence information at all (*n* = 3) (Ziegler et al. 2020) was available. These WNV cases did not meet the required criteria for the APC/AHC grouping, i.e. WNV complete coding sequences with <10 nucleotide gaps or ambiguities. As expected, the available partial WNV genome sequences of the two human cases (MN794936 and MW142225) had the highest sequence identities with members of Subcluster 2.5.3.4.3c. In addition, recently published complete coding sequences (MZ964751.1, MZ964752.1, and MZ964753.1) from three human WNV cases reported in 2021 (Schneider et al. 2021) have the highest sequence identities with members of Subcluster 2.5.3.4.3c. Therefore, as of writing, only members of Subcluster 2.5.3.4.3c have been reported to cause WNV infection in humans in Germany. Members of Subcluster 2.5.3.4.3a, likewise detected in Germany, have previously been reported to cause human WNV infection in other countries, i.e. Austria (Kolodziejek et al. 2015, 2018). The higher spread and frequency of Subcluster 2.5.3.4.3c in Germany are the likely cause for it being the sole subcluster so far associated with human WNV cases reported in Germany.

Here, we also obtained the complete coding sequence of WNV detected in a horse from 2018 (C5), grouping in Subcluster 2.5.3.4.3a (Figs 4 and 7). Viruses of Subcluster 2.5.3.4.3a have been found widespread across Europe over a long period of time, e.g. in Italy (2011), Austria (2015–6), the Czech Republic (2013), Slovakia (2013), Slovenia (2018), Germany (2018–9), and the Netherlands (2020) (Fig. 4). Noteworthy, in 2020, we did not find any member of this geographically widely dispersed subcluster among the sequenced WNV cases. One interesting pair of sequences (MW036634 and LR743448) highlighting this incomplete knowledge is derived from a Culex mosquito pool collected in Utrecht, the Netherlands, in 2020 and from a Humboldt penguin (*Spheniscus humboldti*) collected in Cottbus, Brandenburg, Germany, in 2019, respectively. These WNV cases from Cottbus and Utrecht were detected >600 km apart within roughly 1 year. Given the large distance between Utrecht and Cottbus together with the ubiquitous distribution of Subcluster 2.5.3.4.3a in Europe, we suspect that these two WNV cases might be independent of each other. Despite the fact that according to the phylogenetic trees, they are the closest known relatives, they only have a pairwise identity of 99.67 per cent which is at the lower end of the range of pairwise identities within their subtree, which might imply no direct connection between both. This is in line with the results of the BEAST analysis summarized with TreeAnnotator and visualized in the MCC tree in Fig. 4. As shown there, this analysis estimated the MRCA of the two to have existed around 2013 (HPD 95 per cent: 2011–5 and pp: 35 per cent) (Fig. 4). Altogether, this implies a lack of complete genome sequences from European WNV, as highlighted by the few sequences available for Subcluster 2.5.3.4.3a, which is present for more than 10 years in Southern and Central Europe. In addition, due to only sporadic occurrence of WNV in certain regions where probably the environmental conditions do not permit WNV establishment, like for instance in Northern Germany 2019–20 (Ziegler et al. 2020; Fig. S2), there may be insufficient awareness in the public and among experts. Notwithstanding long-distance translocation of WNV-infected mosquitoes inside vehicles (Brown et al. 2012; Eritja et al. 2017; Bakran-Lebl et al. 2021; Ronca, Ruff, and Murray 2021), long-distance jumps of WNV may rather be implied by undetected cases, both in endemic areas without surveillance and in areas with only sporadic occurrence. Taken together, to fill the gaps we demonstrate, more comprehensive continued genomic surveillance is necessary, not only, as discussed, within Germany but also all over Europe. To achieve this, an overall increased awareness and additional sequencing efforts are essential.

## 4. Conclusions

Here, we introduced a structured and unbiased clustering workflow to systematically allocate WNV complete coding sequences into hierarchical groups below the species level: lineages, clades, subclades, clusters, and subclusters. In addition, we propose a generic hierarchical decimal numbering system designating each group below species rank. We successfully applied the method to allocate WNVs into groups below the species level, and this workflow can also be applied to classify other virus species into hierarchical subgroups. Our workflow only requires a matrix of pairwise sequence identities as input. Essential parameters (e.g. number of clusters, threshold) are entirely decided by the mathematical algorithm, thus removing subjective input from users. Furthermore, the results of our workflow can be combined with different analyses, such as the classical phylogenetic ML tree and the time scaled MCC tree.

Our analyses revealed that Subcluster 2.5.3.4.3c was the predominant WNV subcluster circulating in Germany from 2018 to 2020, accompanied by co-circulating minor WNV (sub)clusters. This finding indicates that the WNV genetic diversity in Germany is primarily influenced by the successful establishment, enzootic maintenance, and expansion of Subcluster 2.5.3.4.3c, possibly supplemented with continuous incursion and potential overwintering of WNV of other (sub)clusters. These other (sub)clusters detected in Germany overlapped in space and time with the dominant Subcluster 2.5.3.4.3c. The minor groups were found in both wild and captive birds, as well as in horses. Therefore, to obtain the full picture of WNV circulation, it will be necessary to obtain whole-genome sequences from all WNV cases whenever possible, to ensure that also minorities are found.

Since all human WNV cases in 2020 occurred in WNV hot spot areas, our study affirmed the importance of birds and horses as sentinels for human WNV infections. Thus, information dissemination regarding WNV infections should be conducted among health-care and veterinary workers and the greater public. Furthermore, we recommend that horses located in these WNV hot spot areas and nearby regions be vaccinated against WNV according to the recommendations of the Standing Committee on Vaccination for Veterinary Medicine in Germany.

## Supplementary Material

vead013_Supp

## Data Availability

The nucleotide sequences from this study are available from the INSDC databases study accession PRJEB47687.
