# Supplementary material for: An advanced sequence clustering and designation workflow reveals the enzootic maintenance of a dominant West Nile virus subclade in Germany
Source: Virus Evol. 2023 Mar 17;9(1):vead013. doi: 10.1093/ve/vead013 (PMC10184446; doi:10.1093/ve/vead013)
Supplement: vead013_Supp [file vead013_supp.zip › suppl_data/SupplementaryMaterial_20230215_komprimiert.docx]

**Supplementary material for**

“An advanced sequence clustering and designation workflow reveals the enzootic maintenance of a dominant West Nile virus subclade in Germany”

Pauline Dianne Santos^1,†^, Anne Günther^1,†^, Markus Keller^2^, Timo Homeier-Bachmann^3^, Martin H. Groschup^2,4^, Martin Beer^1^, Dirk Höper^1,†^, Ute Ziegler^2,4, †,*^

**Contents**

- R-Code

- Figure S1

- Figure S1

- Table S1

- Table S2

- Table S3

- Table S4

**R-Code**

### AP-Clustering of genome sequences based on an identity-matrix

## load necessary R-packages

library(apcluster)

library(writexl)

rm(list = ls()) # clean up workspace to prevent from interference between calculation and pre-existing data

## settings; when multiple values are provided, all possible combinations are tested

minStepNumbers <- 10000 # c(1000, 2000, 5000, 10000) # the minimum number of steps to divide the input preference range for plateau calculations

stepFactor <- 10 # currently not used; Factor to calculate th allowed maximum step number from the used minStepNumbers

windowProportions <- 0.0001 # c(0.01, 0.005, 0.0025, 0.001, 0.0001) # the fraction of the complete steps (see above) the partial dataset used for the APC should have

minPlateauWindows <- 3 # the allowed minimum the actual window may have (values < 3 do not make sense)

groupSizes <- 5 # c(5, 7, 10) # the minimum number of sequences of a subgroup to use as input for a further subgrouping

maxGroupDepth <- 5 # number of hierarchy levels to calculate with 1=Lineage/2=Clade/3=Subclade/4=Cluster/5=Subcluster

## load and prepare input data

workDir <-choose.dir(caption = "please choose the project folder") # choose the folder to read the input from and to save the output

setwd(workDir) # change into the chosen folder

dateipfad <- choose.files(default = paste(workDir, "/*.*", sep = ""), caption = "Please select the identity matrix", multi = FALSE) # choose the input file containing the matrix of sequence identities

sequenceIdentityMatrix <- read.csv(dateipfad, row.names = 1) # read in the matrix of sequence identities

if(identical(colnames(sequenceIdentityMatrix), rownames(sequenceIdentityMatrix))) { # check whether or not the row and column names are identical, if not do not calculate because it might generate invalid results

dissimilarityMatrix <- -1*((1-as.matrix(sequenceIdentityMatrix))^2) # convert the sequence identity matrix into a matrix of dissimilarities as this is the input for affinity propagation clustering

for(aktStepNumber in minStepNumbers) { # iterate the calculations over all preset numbers of steps over the input preference range

for(winProp in windowProportions) { # iterate the calculations over all preset proportions for plateau definition

for(aktGroupSize in groupSizes) { # iterate the calculations over all preset minimum group sizes

for(aktPlateauWindow in minPlateauWindows) { # iterate the calculations over all preset minimum plateau window lengths

dateien <- list.files() # read in a list of files currently present in your target folder

## prepare necessary objects to accomodate results

paramCollection <- data.frame(level = NA, group = NA, inputGroupSize = NA, usedStepWidth = NA, setIterations = NA, usedIterations = NA, minPrefRange = NA, maxPrefRange = NA, terminatingInPref = NA, defaultStepNumber = NA, lastStable = NA, longest = NA, defaultAPC = NA, cutCrit = NA, usedWindowSize = NA) # set up a data.frame to collect all relevant parameters used in the current iteration

allResults <- vector("list", 4) # set up a list to collect all clustering results from the current iteration

names(allResults) <- c("group", "defaultAPresult", "cutreeResult", "aggExResult") # name the elements of the list

## calculate settings adjusted according to above input

maxStepNumber <- aktStepNumber * stepFactor # currently not used; calculate the maximum allowed step number

initSteps <- (aktStepNumber + maxStepNumber) / 2 # currently not used; calculate the initial number of steps to start with

plateauWindow <- floor(aktStepNumber * winProp) # set the window size to fit the actual step number and interrogate always the same portion of the overall range

if(plateauWindow < aktPlateauWindow) plateauWindow <- aktPlateauWindow ## in case the combined settings result in a window smaller than the minimum allowed window size, adjust the setting to the allowed minimum

## prepare filenames to save results

datensatz <- sub("\\.[[:alpha:]]{1,}$", "", basename(dateipfad)) # extract the name of the dataset from the filepath

filename <- paste(datensatz, ".Affiliations-minSteps_", aktStepNumber, "-window_", plateauWindow,

"-minMembers_", aktGroupSize, "-maxGroupDepth_", maxGroupDepth, ".xlsx", sep = "") # construct filename containing the distinguishing parameters

if(is.element(filename, dateien) == FALSE) { # check whether the calculation using the recent parameter combination was already initiated; only if not, continue calculating, otherwise skip to the next combination

write_xlsx(data.frame("Analysis in progress"), filename, col_names = FALSE) # write a file into the current folder to mark the parameter combination in progress

print(paste("Analysis in progress: ", filename, sep = "")) # output user information

seqSubset <- colnames(dissimilarityMatrix) # define the initial set of sequences, i.e. use all sequences of the dataset

affiliations <- data.frame(matrix(nrow = length(seqSubset), ncol = maxGroupDepth + 3)) # set up an object to save the results of the APC; needs 3 more columns than the number of hierarchy levels defined by maxGroupDepth

colnames(affiliations) <- c("accession", "affiliation", paste("affil", 0:maxGroupDepth, sep = "0")) # set the column names

rownames(affiliations) <- colnames(dissimilarityMatrix) # set rownames identical with colnames of the input identity matrix

affiliations$accession <- rownames(affiliations) # copy the rownames to the first column of the result matrix

affiliations$affil00 <- 1 # set the initial affiliation level to 1 for all sequences

affiliations$affiliation <- 0 # set the affiliation 0 for all sequences

for(mgd in 0:(maxGroupDepth - 1)) { # iteratively run through the grouping for all sequences for the given number of hierarchy levels

prevLevel <- paste("affil", mgd, sep = "0") # the previous hierarchy level, the starting point for subsetting the dataset

currLevel <- paste("affil", (mgd + 1), sep = "0") # the current hierarchy level to be determined for the respective subset of sequences

affiliations[, currLevel] <- 0 # set the initial affiliations in the currently analysed hierarchy level to the default value

for(aktSubGroup in unique(affiliations$affiliation[affiliations[,prevLevel] > 0])) { # run the grouping for the current hierarchy level for all subgroups of the preceding level

seqSubset <- affiliations$accession[affiliations$affiliation == aktSubGroup] # generate the list of sequence names belonging to the currently analysed subgroup

if(length(seqSubset) < aktGroupSize) affiliations[seqSubset, currLevel] <- -1 else { # check whether the number of sequences in the current group is sufficient according to the preset minimum group size to allow for further subdivision; if not, set the current affiliation -1 to stop further evaluation in the subsequent iterations

workmat <- as.matrix(dissimilarityMatrix[seqSubset, seqSubset]) # if the current group size allows for further subdivision, get the working matrix only containing data of the relevant subset

workmatAPC <- apcluster(workmat, details=TRUE, q=0.5, nonoise = TRUE) # calculate the AP-clustering of the current data subset using the default input preference q

prefRang <- preferenceRange(workmat, exact=TRUE) # determine the input preference range of the data subset

pStepWidth <- abs((prefRang[2] - prefRang[1]) / (aktStepNumber - 2)) # adjust the step width to cover the complete input preference range in equal steps

inPref <- unique(c(prefRang[1], seq(prefRang[1], prefRang[2], pStepWidth), prefRang[2])) # calculate all input preferences to use in the APC iterations

plateauWindow <- floor(length(inPref) * winProp) # set the window size to fit the actual step number and interrogate always the same portion of the overall range

if(plateauWindow < aktPlateauWindow) plateauWindow <- aktPlateauWindow # in case the calculated window size for the determination of a cluster number plateau is lower than the preset lower level, adjust the size of the window used to define the plateau to fit with the lower limit

if(length(inPref) > plateauWindow & length(workmatAPC@clusters) > 2) { # test whether sufficient iterations are performed to cover the set window for plateau determination, only start iterating if yes because otherwise an error will occur

clusTab <- data.frame(inPref, numClust = NA, windowStDev = NA, windowMean = NA, increase = TRUE, stDevOK = TRUE) # prepare table to save the results of all clustering iterations to enable testing whether or not the stopping criteria are met

i <- 0 # define counter

stopAPC <- FALSE # set the control variable

while(i < nrow(clusTab) & stopAPC == FALSE) { # repeat the calculations for AP clustering of the current data subset until either of the stopping criteria is met

i <- i + 1 # increase counter for current iteration

j<-apcluster(workmat, p = clusTab$inPref[i], nonoise = TRUE) # determine the number of AP clusters in the data subset with the given input preference (as previously defined from the preference range and chosen number of iterations)

clusTab$numClust[i] <- length(j@clusters) # record the number of AP clusters corresponding with the input preference

if(i <= plateauWindow) { # check whether or not sufficient data for calculation of mean and SD from number of clusters is available

if(i == 1) clusTab$windowStDev[i] <- 0 else clusTab$windowStDev[i] <- sd(clusTab$numClust[1:i]) # if not, set SD of cluster number within window 0 in case of first iteration, otherwise adjust SD calculation to available data instead of preset window

clusTab$windowMean[i] <- mean(clusTab$numClust[1:i]) # calculate mean from the available data

} else { # number of performed iterations higher than window size for plateau definition

clusTab$windowStDev[i] <- sd(clusTab$numClust[(i - plateauWindow + 1):i]) # calculate SD of cluster number from recent and preceding iterations (in total preset number of iterations)

clusTab$windowMean[i] <- mean(clusTab$numClust[(i - plateauWindow + 1):i]) # calculate mean cluster number from recent and preceding iterations (in total preset number of iterations)

clusTab$increase[i] <- clusTab$numClust[i] >= clusTab$numClust[(i-1)] # test whether the number of clusters is the same as or larger than in the preceding iteration, because a decrease is deemed a disruption and leads to termination of the iterative AP clustering

tempTab <- clusTab[(i - plateauWindow + 1):i,] # make subset of the table only containing data of the plateauWindow number of rows including the last iteration

clusTab$stDevOK[i] <- nrow(tempTab[tempTab$windowStDev != 0,]) < plateauWindow # test whether the SD of the cluster number returns to 0 after an increase of the cluster number (this must be the case if the cluster number is stable for at least plateauWindow iterations), if not a disruption occurred

stopAPC <- !(clusTab$increase[i] == TRUE & clusTab$stDevOK[i] == TRUE) # check whether or not both criteria to enter the next iteration, i.e. not to terminate the loop, are fulfilled

}

}

clusTab$valid <- FALSE # set a flag for selection of the lines containing valid plateau data

if(clusTab$stDevOK[i] == TRUE & clusTab$increase[i] == FALSE) { # check the reason to stop the iterative ap-clustering; if there is a decrease in cluster number without the standard deviation being != 0 for more than the window size, then determine the cluster number of the current plateau needs to be determined

destabNumClust <- min(clusTab$numClust[(i - plateauWindow):(i - 1)]) # in case a decrease in cluster number occurs, the plateau within which the decrease occurs, is disrupted, therefore, the cluster number preceding the current step needs to be determined and all iterations resulting in this cluster number need to be removed as disrupted plateau

clusTab$valid[clusTab$numClust < destabNumClust] <- TRUE # all plateaus preceding the disrupted plateau (i.e. with numbers lower than the disrupted) are valid

}

if(clusTab$stDevOK[i] == FALSE) clusTab$valid[1:(i - plateauWindow)] <- TRUE # in case the cluster number does not decrease but the standard deviation != 0 for too many iterations led to stopping the calculations, then all plateaus preceding the point of disruption for at least the window size are valid

clusTab$valid[clusTab$increase == FALSE] <- FALSE # a cluster number that is lower than the cluster number in the preceding iteration must always be set invalid

clusTab$valid[1:(plateauWindow - 1)] <- FALSE # the starting iterations with numbers smaller than the set minimum window size must be set invalid, because they do not constitute a complete window, i.e., they cannot be used alone

# assign(paste("clusTab", aktSubGroup, sep = "."),value = clusTab) # optionally activate by removing the "#" at the line beginning to save results

setIterations <- nrow(clusTab) # record the set maximum number of iterations

usedIterations <- nrow(clusTab[!is.na(clusTab$numClust), ]) # record the number of performed iterations

firstPlateau <- clusTab$numClust[1] # the first plateau is always the first number of clusters found in the iterations

clusTab$valid[1:min(which(diff(clusTab$numClust) != 0))] <- FALSE # invalidate the iterations corresponding to the first plateau as per the definition in Susanne Fischer's paper the first plateau is not valid

plateauSummary <- clusTab$numClust[clusTab$valid == TRUE] # prepare the identification of the longest plateau

plateauSummary <- summary(as.factor(plateauSummary), maxsum = length(unique(plateauSummary))) # summarize how often each number of clusters was observed to define the longest plateau, i.e. the number of clusters that was most often observed before the disruption

if(length(plateauSummary) > 0) { # test whether any valid plateau was detected and if yes determine the values of the last and longest plateau

longestPlateau <- max(as.numeric(names(plateauSummary[plateauSummary == max(plateauSummary)]))) # determine the number of clusters constituting the longest plateau; in case 2 or more cluster numbers are present the same number of iterations, use the higher number of clusters in order not to reduce the cluster number too stringently

lastStable <- max(as.numeric(names(plateauSummary))) # determine the last stable plateau, which is the highest number occurring in the names of the plateau summary, because the invalid plateaus were removed prior to determination of the plateau summary

} else { # in case no valid plateau was found, set the empty vectors to enable the following calculations

longestPlateau <- integer()

lastStable <- integer()

}

allPlateaus <- c(lastStable, longestPlateau) # concatenate the determined cluster numbers from the longest and the last stable plateau

if(is.element(TRUE, allPlateaus < length(workmatAPC@clusters))) { # define the best number of clusters to use and record the used choice; the best choice is the highest number of clusters that is equal or lower than the number of clusters determined with the default input preference, therefore, test whether either the last stable or the longest plateau are more stringent than the default

cutNum <- max(allPlateaus[allPlateaus < length(workmatAPC@clusters)]) # record the number of clusters to use for cutting the tree (below)

if(cutNum == lastStable) cutCrit <- "last" else cutCrit <- "longest" # record the choice in case the default is replaced

} else { # the default value is used

cutNum <- length(workmatAPC@clusters) # record the default value of the cluster number to use it for tree cutting below

cutCrit <- "defaultAPC" # record the used choice

}

}

if(length(workmatAPC@clusters) <= 2) { # in case only 1 or 2 clusters are defined using the default settings, use the default value

cutNum <- length(workmatAPC@clusters) # record the default value of the cluster number to use it for tree cutting below

cutCrit <- "defaultAPC" # record the used choice

}

aggdissimilarityMatrix <- aggExCluster(workmat, workmatAPC) # agglomerative hierarchical clustering

grouping <- cutree(aggdissimilarityMatrix, k = cutNum) # cutting the tree to determine the resulting grouping of sequences; k = number of groups to have = cluster number as determined above

if(length(grouping@clusters) == 1) for(g in 1:length(grouping@clusters)) affiliations[rownames(workmat)[grouping@clusters[[g]]], currLevel] <- -3 else for(g in 1:length(grouping@clusters)) affiliations[rownames(workmat)[grouping@clusters[[g]]], currLevel] <- g # record the grouping in the current subset of the current hierarchical level; in case the subset cannot be further subdivided (number of clusters is 1), record -3 to label the grouping being terminated for the subset because it cannot be further subdivided; in all other cases, record the group affiliations per sequence

if(length(inPref) <= plateauWindow) affiliations[seqSubset, currLevel] <- -2 # in case there is not enough steps for the calculations, report -2 to label the subgroup for subsequent cycles and error analysis

## in the following lines, record all current settings of the iteration

if(length(workmatAPC@clusters) <= 2) { # record the available values in case no iterative calculation was done because the default was too low

paramCollection$group[nrow(paramCollection)] <- aktSubGroup

paramCollection$level[nrow(paramCollection)] <- mgd

paramCollection$inputGroupSize[nrow(paramCollection)] <- length(seqSubset)

paramCollection$defaultAPC[nrow(paramCollection)] <- length(workmatAPC@clusters)

paramCollection$cutCrit[nrow(paramCollection)] <- "defaultAPC"

paramCollection <- rbind(paramCollection, NA) # add the next line to the table to accommodate the data of the next iteration

} else { # record all relevant data in case the iterative calculation for plateau determination was done

paramCollection$terminatingInPref[nrow(paramCollection)] <- clusTab$inPref[usedIterations]

paramCollection$level[nrow(paramCollection)] <- mgd

paramCollection$group[nrow(paramCollection)] <- aktSubGroup

paramCollection$inputGroupSize[nrow(paramCollection)] <- length(seqSubset)

paramCollection$usedStepWidth[nrow(paramCollection)] <- pStepWidth

paramCollection$setIterations[nrow(paramCollection)] <- setIterations

paramCollection$usedIterations[nrow(paramCollection)] <- usedIterations

paramCollection$minPrefRange[nrow(paramCollection)] <- prefRang[1]

paramCollection$maxPrefRange[nrow(paramCollection)] <- prefRang[2]

if(length(lastStable) > 0) paramCollection$lastStable[nrow(paramCollection)] <- lastStable else paramCollection$lastStable[nrow(paramCollection)] <- NA

if(length(longestPlateau) > 0) paramCollection$longest[nrow(paramCollection)] <- longestPlateau else paramCollection$longest[nrow(paramCollection)] <- NA

paramCollection$defaultAPC[nrow(paramCollection)] <- length(workmatAPC@clusters)

paramCollection$cutCrit[nrow(paramCollection)] <- cutCrit

paramCollection$usedWindowSize[nrow(paramCollection)] <- plateauWindow

paramCollection <- rbind(paramCollection, NA) # add the next line to the table to accommodate the data of the next iteration

}

}

## End parameter recording

## in the following lines, record all results of the current iteration

allResults$group <- append(allResults$group, aktSubGroup)

allResults$defaultAPresult <- append(allResults$defaultAPresult, workmatAPC)

allResults$aggExResult <- append(allResults$aggExResult, aggdissimilarityMatrix)

allResults$cutreeResult <- append(allResults$cutreeResult, grouping)

## End results recording

}

if(mgd == 0) affiliations$affiliation[affiliations[, currLevel] > 0] <- affiliations[affiliations[, currLevel] > 0, currLevel] else affiliations$affiliation[affiliations[, currLevel] > 0] <- paste(affiliations$affiliation[affiliations[, currLevel] > 0], affiliations[affiliations[, currLevel] > 0, currLevel], sep = ".") # construct the overall group designation from the previously present portion and the currently analyzed hierarchy level; in case it is the first level iteration, replace the present values with the current

}

affiliations$affil00 <- NULL # delete the initial grouping

## save results to disk

write_xlsx(affiliations, filename)

write_xlsx(paramCollection, sub("Affiliations", "usedParameters", filename))

save.image(file = sub("Affiliations", "CompleteData", sub("xlsx", "RData", filename)))

}

}

}

}

}

} else print("Please check the column and row names in your input file! They must be identical!")

**Figure S1.** Geographic distribution of WNV cases in Germany in 2020 (depicted on district level) as shown in A. Specific areas with WNV cases in the areas of Saxony, Saxony-Anhalt, Thuringia, Berlin and Brandenburg were shown in B and WNV cases in Berlin and surrounding areas in Brandenburg were shown in C. Blue squares and red circles indicate notifiable WNV cases of horses and birds. WNV cases with numbers indicated that these samples were subjected to whole-genome sequencing. WNV cases that were not selected for sequencing (e.g., IgM-positive cases or high C_q_ values) remain unnumbered. Intensity of the colored background at district level indicates the frequency, how often an area was affected by WNV activity in prior years.


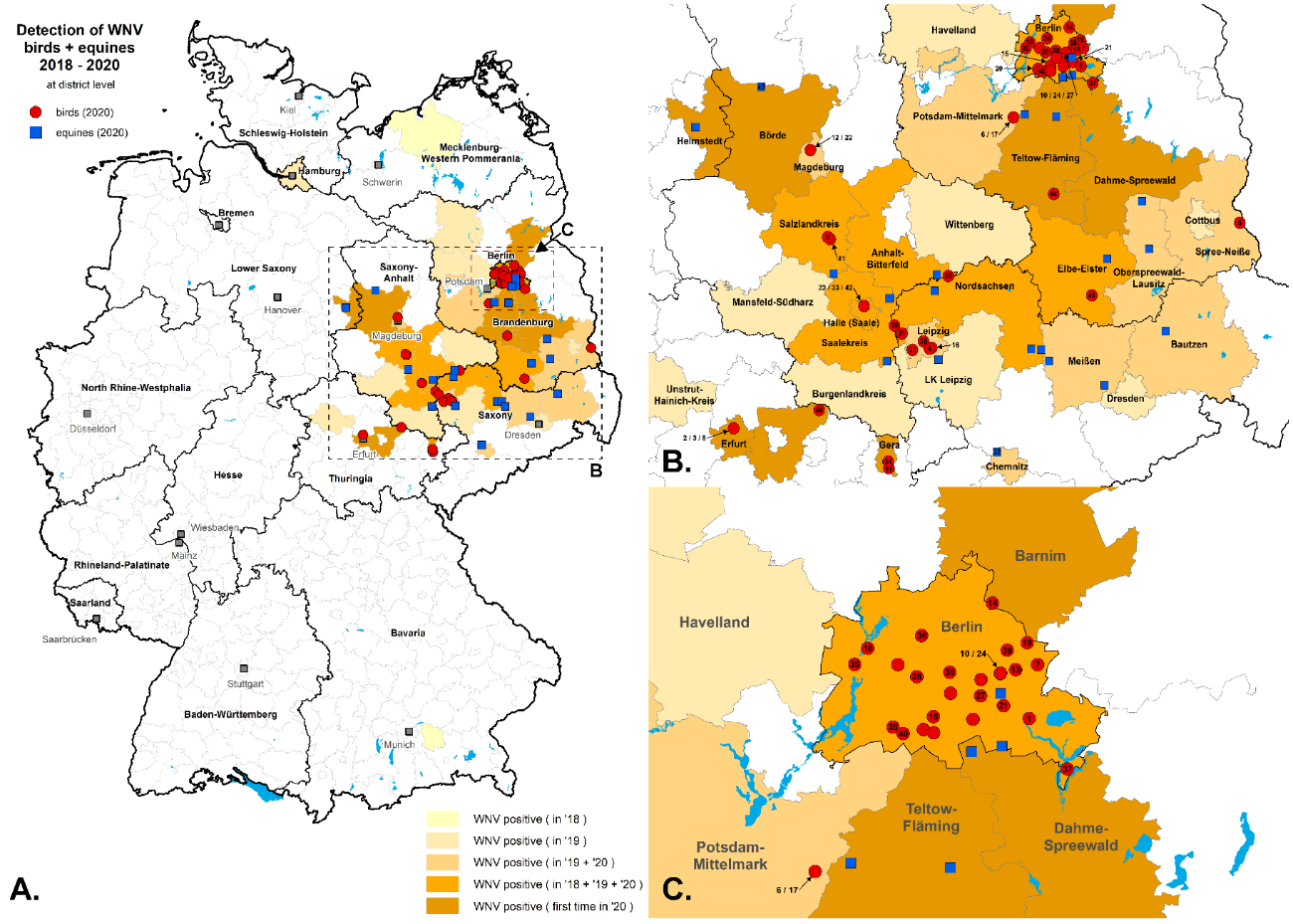


**Figure S2** Climatological maps of Germany displaying A) temperature (in degree celsius), B) precipitation (in millimeter) and C) water balance (in millimeter) based on data collected in summer 2020. Climatological maps were downloaded from Deutscher Wetterdienst; <https://www.dwd.de/EN/climate_environment/climatemonitoring/germany/germany_node.html> (Deutscher Wetterdienst 2020)


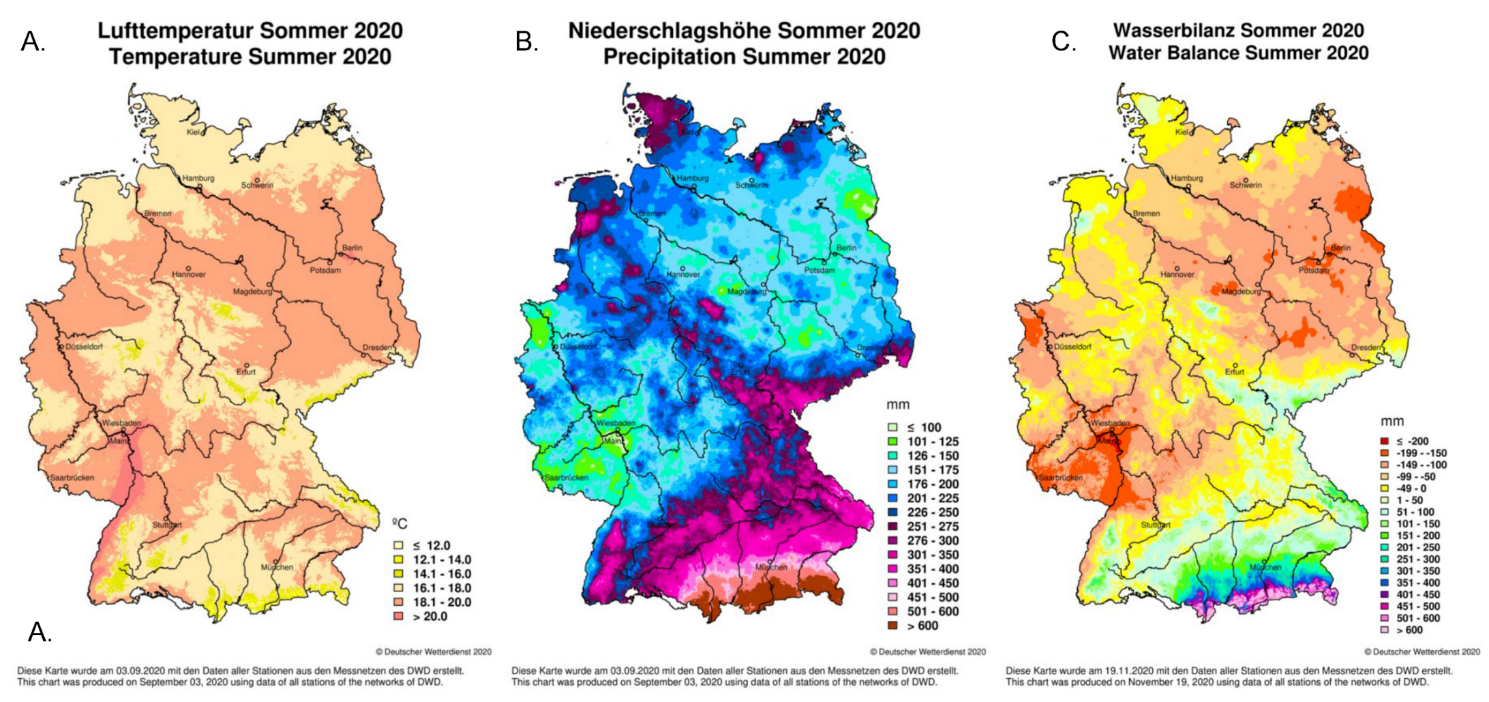


Deutscher Wetterdienst (2021), 'Climatological maps of Germany', <https://www.dwd.de/EN/ourservices/klimakartendeutschland/klimakartendeutschland.html?nn=495490>, accessed 15.07.2021.

**Table S1** West Nile Virus primer sequences from Sikkema et al. 2020. Each primer stock was normalized to 100 micromolar concentration. Volumes of each primer per primer mix (mix 1 or 2) were specified in the table below. These primers mixes were then subjected to 1:10 dilution.

| **Primer number** | **Mix** | **Primer** | **Primer sequence** | **Used volume (µl)** |
| --- | --- | --- | --- | --- |
| 1 | 1 | WNVUS1_1_LEFT | GCCTGTGTGARCTGACAAACTTAG | 10 |
| 2 | 1 | WNVUS1_1_RIGHT | CTTTTCTTTTGTTTTGRGCTCCG | 10 |
| 3 | 1 | WNVUS1_3_LEFT | AGTTACCCTCTCTAACTTCCAAG | 15 |
| 4 | 1 | WNVUS1_3_LEFT_2 | GTGACCCTCTCCAACTTCCAGG | 15 |
| 5 | 1 | WNVUS1_3_RIGHT | CARGAAGTCTCTGTTRCTCATTCC | 15 |
| 6 | 1 | WNVUS1_5_LEFT_2 | GTGTCCAACCATGGGTGAAGCC | 10 |
| 7 | 1 | WNVUS1_5_LEFT | GCCCGACCATGGGAGAAGCT | 10 |
| 8 | 1 | WNVUS1_5_RIGHT_2 | GTGGCATGAGGTTCTTCAAACTCC | 10 |
| 9 | 1 | WNVUS1_5_RIGHT | GGCGTGTGGTTCCTCAAACTCC | 10 |
| 10 | 1 | WNVUS1_7_LEFT_2 | TCTGAAGTGTAGGGTGAAGATGGAG | 10 |
| 11 | 1 | WNVUS1_7_LEFT | GTCATTTGAAGTGTAGAGTGAAGATGG | 10 |
| 12 | 1 | WNVUS1_7_RIGHT | GAGGTGAAMACCCCTCCAACTG | 10 |
| 13 | 1 | WNVUS1_9_LEFT | GTGGATGGGMATCAATGCYCGT | 10 |
| 14 | 1 | WNVUS1_9_RIGHT_2 | CTCTTGCCCCAAGCCTTCCAAC | 10 |
| 15 | 1 | WNVUS1_9_RIGHT | CTTTCCCCAGGCCTTCCAGC | 10 |
| 16 | 1 | WNVUS1_11_LEFT | CACAACKGAATGYGACTCGAAGAT | 10 |
| 17 | 1 | WNVUS1_11_RIGHT | ACGGTGTCCGCAGCTCTCAC | 10 |
| 18 | 1 | WNVUS1_11_RIGHT_2 | CACGGTGTCCGCAACTRTCAC | 10 |
| 19 | 1 | WNVUS1_13_LEFT_2 | GGCACGACGAAAAGACCCTCGTGC | 10 |
| 20 | 1 | WNVUS1_13_LEFT | GACATGATGAAAAGACCCTCGTGC | 10 |
| 21 | 1 | WNVUS1_13_RIGHT_2 | CTCTTGGTTGGTCCACCTTGC | 10 |
| 22 | 1 | WNVUS1_13_RIGHT | CTCCTGGTTGGTCCATCTCGC | 10 |
| 23 | 1 | WNVUS1_15_LEFT_2 | CAAATGTGGTGGTGCCGCTGC | 10 |
| 24 | 1 | WNVUS1_15_LEFT | CGACATCAAACGTGGTTGTTCCG | 10 |
| 25 | 1 | WNVUS1_15_RIGHT_2 | CYGTCCTCTCAATCCACATGTC | 10 |
| 26 | 1 | WNVUS1_15_RIGHT | CGCCGTTCTCTCAATCCACATATC | 10 |
| 27 | 1 | WNVUS1_17_LEFT_2 | ATAAGTGCCTACACACCYTGGGC | 10 |
| 28 | 1 | WNVUS1_17_LEFT | GGAARATATGGATGCTCAGAATGG | 10 |
| 29 | 1 | WNVUS1_17_RIGHT_2 | CCCCAATTTCTCCTTCTGGTGTC | 10 |
| 30 | 1 | WNVUS1_17_RIGHT | TTTGAACACCCCTGGTTTCGTC | 10 |
| 31 | 1 | WNVUS1_19_LEFT_2 | CCATTGTGCAAGGAGAGAGAATGG | 10 |
| 32 | 1 | WNVUS1_19_LEFT | CGGATTCGAACCTGAGATGCTG | 10 |
| 33 | 1 | WNVUS1_19_RIGHT_2 | CGATGCTCGCTGGATCCGTG | 10 |
| 34 | 1 | WNVUS1_19_RIGHT | CATGAATATTGCCGCCGCCTC | 10 |
| 35 | 1 | WNVUS1_21_LEFT_2 | GGAAAGACCGTTTGGTTTGTTCC | 10 |
| 36 | 1 | WNVUS1_21_LEFT | GGGAAGACGGTTTGGTTTGTGC | 10 |
| 37 | 1 | WNVUS1_21_RIGHT_2 | GAGTCGTCTTCATTCGTGTGCC | 10 |
| 38 | 1 | WNVUS1_21_RIGHT | GTTGGAATCATCCTCATTTGTGTGC | 10 |
| 39 | 1 | WNVUS1_23_LEFT | CGGCTGGAGTGTCATACCACG | 10 |
| 40 | 1 | WNVUS1_23_LEFT_2 | CAGCAGGAATATCATACCATGACC | 10 |
| 41 | 1 | WNVUS1_23_RIGHT_2 | CTATTGTCTGAAGGGCGTCCGG | 10 |
| 42 | 1 | WNVUS1_23_RIGHT | GAATACTCCCATGGTCATCACACTC | 10 |
| 43 | 1 | WNVUS1_25_LEFT_2 | CAGGAACGAAAATAGCAGGCATGC | 10 |
| 44 | 1 | WNVUS1_25_LEFT | GGAACGAAGATCGCCGGAATG | 10 |
| 45 | 1 | WNVUS1_25_RIGHT_2 | GCTTCCGCTTGCCAGCCTG | 10 |
| 46 | 1 | WNVUS1_25_RIGHT | GCTGAGCGCATTGCCTCAGC | 10 |
| 47 | 1 | WNVUS1_27_LEFT_2 | CAGTCATGCAGAAAAARGTTGGACAG | 10 |
| 48 | 1 | WNVUS1_27_LEFT | GATCTTGGTGTCTCTAGCTGCAG | 10 |
| 49 | 1 | WNVUS1_27_RIGHT_2 | CGAGATCCACAACCTTTCCCAC | 10 |
| 50 | 1 | WNVUS1_27_RIGHT | CATCCAAGGTCAATCACTTTTCCG | 10 |
| 51 | 1 | WNVUS1_29_LEFT_2 | CTGGCCATGAAGAGCCACAAC | 10 |
| 52 | 1 | WNVUS1_29_LEFT | GTACAGGAAGTGAAAGGGTACACG | 10 |
| 53 | 1 | WNVUS1_29_RIGHT_2 | GTTGACATCTTCCTCAAACTGGGG | 10 |
| 54 | 1 | WNVUS1_29_RIGHT | GCCCTGGTTCCACTTCCCAAG | 10 |
| 55 | 1 | WNVUS1_31_LEFT_2 | GAATACAGCTCCACATGGCACC | 10 |
| 56 | 1 | WNVUS1_31_LEFT | GAGAACCACCCATATAGAACCTGG | 10 |
| 57 | 1 | WNVUS1_31_RIGHT | CTCTTTCCCATCATGTTGTARATGC | 10 |
| 58 | 1 | WNVUS1_33_LEFT_2 | GGGTACATCTTGAAGGAAGTYGG | 10 |
| 59 | 1 | WNVUS1_33_LEFT | GTTACATCCTGCGTGAAGTTGGC | 10 |
| 60 | 1 | WNVUS1_33_RIGHT | CSCCATTCTCAAACAGCCAGG | 10 |
| 61 | 1 | WNVUS1_35_LEFT_2 | GGTGGTATGACTGGCAGCAGG | 10 |
| 62 | 1 | WNVUS1_35_LEFT | GATGGTATGATTGGCAGCAGGTTC | 10 |
| 63 | 1 | WNVUS1_35_RIGHT | GTCTTCCATCCAYTCATTCTCCTC | 10 |
| 64 | 1 | WNVUS1_37_LEFT | GAGAAGTATGYGGATTACATGAGYTC | 15 |
| 65 | 1 | WNVUS1_37_RIGHT | GGTCTCCTCTAACCTCTAGTCC | 15 |
| 66 | 2 | WNVUS1_2_RIGHT_2 | CGGGCTGTCAATATGCTAAAACGC | 10 |
| 67 | 2 | WNVUS1_2_RIGHT | GTGCACCAGCAGTCAATGTCTTC | 10 |
| 68 | 2 | WNVUS1_2_LEFT | GTGCACCAACAGTCGATGTCTTC | 10 |
| 69 | 2 | WNVUS1_4_LEFT | GGATGCTAGGAAGCAACACAATGC | 10 |
| 70 | 2 | WNVUS1_4_RIGHT_2 | GATGCTTGGRAGCAACACCATG | 10 |
| 71 | 2 | WNVUS1_4_RIGHT | TGCTYCCCTTTCCAAACAGTCC | 10 |
| 72 | 2 | WNVUS1_4_LEFT_2 | GCTTCCTTTGCCAAATAGTCCGC | 10 |
| 73 | 2 | WNVUS1_6_LEFT | GACTGTGARCCACGGTCAGG | 10 |
| 74 | 2 | WNVUS1_6_RIGHT_2 | CCGGTGTATTGCAGTTCCAACAC | 10 |
| 75 | 2 | WNVUS1_6_RIGHT | GCAATTCCAACACCACAGTGCC | 10 |
| 76 | 2 | WNVUS1_8_LEFT_2 | GTGAATCCATTTGTGTCTGTGGCC | 10 |
| 77 | 2 | WNVUS1_8_LEFT | GTCAACCCTTTTGTTTCAGTGGCC | 10 |
| 78 | 2 | WNVUS1_8_RIGHT_2 | GATCCATCCAGGCTTCCACATC | 10 |
| 79 | 2 | WNVUS1_8_RIGHT | GGTCCATCCAAGCCTCCACATC | 10 |
| 80 | 2 | WNVUS1_10_LEFT_2 | AGACTCGAGCACCAAATGTGGG | 10 |
| 81 | 2 | WNVUS1_10_LEFT | CCAGACTGGAGCATCAAATGTGG | 10 |
| 82 | 2 | WNVUS1_10_RIGHT | GAACYGCCCTYTCAAGCTTCC | 10 |
| 83 | 2 | WNVUS1_12_LEFT | GAAGTYAAATCATGYACSTGGCC | 10 |
| 84 | 2 | WNVUS1_12_RIGHT | CTTGCGAAGGACCTCCTGGG | 10 |
| 85 | 2 | WNVUS1_14_LEFT_2 | GTCCTAGTGTTTGGGGGTATTACG | 10 |
| 86 | 2 | WNVUS1_14_LEFT | CCTGGTGTTTGGGGGCATTAC | 10 |
| 87 | 2 | WNVUS1_14_RIGHT_2 | GCAGATGAGGCAAGCYCCTTTC | 10 |
| 88 | 2 | WNVUS1_14_RIGHT | CAAGCATARCAGACTTGCTCCTTTC | 10 |
| 89 | 2 | WNVUS1_16_LEFT_2 | CTGCAGTTGGACTCATGTTTGCC | 10 |
| 90 | 2 | WNVUS1_16_LEFT | GCTGTCGGCYTRATGTTTGCCA | 10 |
| 91 | 2 | WNVUS1_16_RIGHT_2 | GGTGATGGTGTGTCCCAAAGRAC | 10 |
| 92 | 2 | WNVUS1_16_RIGHT | GAGGGAGTGTCCCACARCAC | 10 |
| 93 | 2 | WNVUS1_18_LEFT_2 | CCACACACTATGGCACACCAC | 10 |
| 94 | 2 | WNVUS1_18_LEFT | GCAGGAGCRGGCGTGATG | 10 |
| 95 | 2 | WNVUS1_18_RIGHT_2 | CTCARTCTTTTGTTGATGGCCTCC | 10 |
| 96 | 2 | WNVUS1_18_RIGHT | GCCACAGATCATCAAAGAGGCC | 10 |
| 97 | 2 | WNVUS1_20_LEFT_2 | GATGTCTCCACACAGAGTCCC | 10 |
| 98 | 2 | WNVUS1_20_LEFT | GATGTCTCCTCACAGGGTGCC | 10 |
| 99 | 2 | WNVUS1_20_RIGHT_2 | GAAAGTCGTAYGAGACGGAGTAC | 10 |
| 100 | 2 | WNVUS1_20_RIGHT | GGGTACTCTGTCTCATAGGACTTTC | 10 |
| 101 | 2 | WNVUS1_22_LEFT_2 | GCTCAGCGGAGAGGACGC | 10 |
| 102 | 2 | WNVUS1_22_LEFT | CGCCCAGAGACGTGGACG | 10 |
| 103 | 2 | WNVUS1_22_RIGHT_2 | CTTTCTCTCACCCAACTTCGTG | 10 |
| 104 | 2 | WNVUS1_22_RIGHT | GGCCTCAGAATCTTCCTTTCACC | 10 |
| 105 | 2 | WNVUS1_24_LEFT_2 | GATCACAAATCGGGCTCGTTGAG | 10 |
| 106 | 2 | WNVUS1_24_LEFT | CGTTCTCAGATAGGGCTCATTGAG | 10 |
| 107 | 2 | WNVUS1_24_RIGHT_2 | CAACTCCCAGRGTCGTCTCTC | 10 |
| 108 | 2 | WNVUS1_24_RIGHT | CTCCTTGACCTCAATTCTTTGCCC | 10 |
| 109 | 2 | WNVUS1_26_LEFT_2 | GTGGACGTTGGTGTGTCAGCTC | 10 |
| 110 | 2 | WNVUS1_26_LEFT | CTTCGTCGATGTTGGAGTGTCG | 10 |
| 111 | 2 | WNVUS1_26_RIGHT_2 | GTTGCATTCCACACTGAACTAGC | 10 |
| 112 | 2 | WNVUS1_26_RIGHT | CCAAACAGAGCTTGCTCCATTCTC | 10 |
| 113 | 2 | WNVUS1_28_LEFT_2 | GGGAAGTTTGGAAGGAGAGACTC | 10 |
| 114 | 2 | WNVUS1_28_LEFT | GTACCGCAAAGAGGCCATCATC | 10 |
| 115 | 2 | WNVUS1_28_RIGHT_2 | CCAATGTCACAGAGCAGTGTGTC | 10 |
| 116 | 2 | WNVUS1_28_RIGHT | GARGACTCTCCGATGTCACAAAG | 10 |
| 117 | 2 | WNVUS1_30_LEFT_2 | CCATGAGATGTACTGGGTGAGY | 15 |
| 118 | 2 | WNVUS1_30_LEFT | GACTGGTCAGAAACCCACTCTC | 15 |
| 119 | 2 | WNVUS1_30_RIGHT_2 | GAAGGGAGTAGTGTCAGTCATGG | 15 |
| 120 | 2 | WNVUS1_30_RIGHT | CACTCGTTGTTGACCGAAAGGAG | 15 |
| 121 | 2 | WNVUS1_32_LEFT_2 | GGAAGAACGCCCGGGAAGC | 10 |
| 122 | 2 | WNVUS1_32_LEFT | GAGGAGCGCCAGAGARGCAG | 10 |
| 123 | 2 | WNVUS1_32_RIGHT_2 | CAGCAGTTCAAGAACCTTCGCTTC | 10 |
| 124 | 2 | WNVUS1_32_RIGHT | CCAAGTCAGCTCTCGTGATGCG | 10 |
| 125 | 2 | WNVUS1_34_LEFT_2 | GTGAAAGTGATGCGCCCGGC | 10 |
| 126 | 2 | WNVUS1_34_LEFT | GTCGTGAAAGTGATGAGGCCAG | 10 |
| 127 | 2 | WNVUS1_34_RIGHT_2 | GAGAWATGCGAGCTCTGCCTAC | 10 |
| 128 | 2 | WNVUS1_34_RIGHT | CGCACATTCCATCCAGCCCC | 10 |
| 129 | 2 | WNVUS1_36_LEFT_2 | CGCAAAAGGAGAATGGATGACGAC | 10 |
| 130 | 2 | WNVUS1_36_LEFT | CCATGCAGGAGGAGAGTGGATG | 10 |
| 131 | 2 | WNVUS1_36_RIGHT | CGTCTACTCAACTTCCGGTGG | 10 |
| 132 | 2 | WNVUS1_38_LEFT | CCCTCAGAACCGTCTCGGAAG | 10 |
| 133 | 2 | WNVUS1_38_RIGHT | GCACTGTGCCGTGTGGCTG | 10 |

**Table S2** Samples used to validate the WNV multiplex PCR High-throughput sequencing (HTS) in comparison with the result of unbiased and direct HTS approach.

| Sample | | |  | Reference sequence | | | |  | This study | | | Result of sequence comparison |
| --- | --- | --- | --- | --- | --- | --- | --- | --- | --- | --- | --- | --- |
| Code | ID | Cq value |  | Library ID | applied protocol | INSDC Accesssion | Length (nt) |  | Library ID | INSDC Accesssion | Length (nt) |  |
| C1 | ED-I-62/19 | 22.9 |  | lib03378 | Wylezich et al 2018 | LR743425 | 11,060 |  | lib04562 | XX | 10,989 | identical |
| C2 | ED-I-156/19 | 11.8 |  | lib03418 | Wylezich et al 2018 | LR743423 | 11,027 |  | lib04563 | XX | 10,989 | Identical |
| C3 | ED-I-155/19 | 17.5 |  | lib03420 | Wylezich et al 2018 | LR743422 | 11,010 |  | lib04564 | XX | 10,987 | Identical |
| C4 | ED-I-115/19 | 31.5 |  | lib03988 | Wylezich et al 2021 | LR989891 | 6,470 |  | lib04565 | XX | 10,989 | 8 substitutions^1^ |
| C5 | ED-I-127-18 | 29.4 |  | lib03224 | Wylezich et al 2021 | Unpublished | 6,786 |  | lib04748 | XX | 10,988 | 2 substitutions^1^ |

^1^ based on available partial reference sequence; insertions and deletions not considered

**Table S3** List of full genome sequences retrieved from Genbank

| **Accession number** | **Used in Dataset** |
| --- | --- |
| DQ116961 | WL2 |
| HQ537483 | WL2 |
| JN858070 | WL2 |
| KC407673 | WL2 |
| KC496015 | WL2 |
| KC496016 | WL2 |
| KF179639 | WL2 |
| KF179640 | WL2 |
| KF588365 | WL2 |
| KF647249 | WL2 |
| KF647250 | WL2 |
| KF647251 | WL2 |
| KF647252 | WL2 |
| KF823806 | WL2 |
| KJ577738 | WL2 |
| KJ577739 | WL2 |
| KJ883342 | WL2 |
| KJ883343 | WL2 |
| KJ883344 | WL2 |
| KJ883345 | WL2 |
| KJ883346 | WL2 |
| KJ883348 | WL2 |
| KJ883349 | WL2 |
| KJ883350 | WL2 |
| KM203860 | WL2 |
| KM203861 | WL2 |
| KM203862 | WL2 |
| KM203863 | WL2 |
| KM659876 | WL2 |
| KP109691 | WL2 |
| KP109692 | WL2 |
| KP780837 | WL2 |
| KP780838 | WL2 |
| KP780839 | WL2 |
| KP789953 | WL2 |
| KP789954 | WL2 |
| KP789955 | WL2 |
| KP789956 | WL2 |
| KP789957 | WL2 |
| KP789958 | WL2 |
| KP789959 | WL2 |
| KP789960 | WL2 |
| KT207792 | WL2 |
| KT359349 | WL2 |
| KT757318 | WL2 |
| KT757319 | WL2 |
| KT757320 | WL2 |
| KT757321 | WL2 |
| KT757322 | WL2 |
| KT757323 | WL2 |
| KU206781 | WL2 |
| KU573080 | WL2 |
| KU573081 | WL2 |
| KU573082 | WL2 |
| KU573083 | WL2 |
| KX375812 | WL2 |
| KY594040 | WL2 |
| LR743421 | WL2 |
| LR743422 | WL2 |
| LR743423 | WL2 |
| LR743424 | WL2 |
| LR743425 | WL2 |
| LR743426 | WL2 |
| LR743427 | WL2 |
| LR743428 | WL2 |
| LR743429 | WL2 |
| LR743430 | WL2 |
| LR743431 | WL2 |
| LR743432 | WL2 |
| LR743433 | WL2 |
| LR743434 | WL2 |
| LR743435 | WL2 |
| LR743436 | WL2 |
| LR743437 | WL2 |
| LR743442 | WL2 |
| LR743443 | WL2 |
| LR743444 | WL2 |
| LR743445 | WL2 |
| LR743446 | WL2 |
| LR743447 | WL2 |
| LR743448 | WL2 |
| LR743449 | WL2 |
| LR743450 | WL2 |
| LR743451 | WL2 |
| LR743452 | WL2 |
| LR743453 | WL2 |
| LR743454 | WL2 |
| LR743455 | WL2 |
| LR743456 | WL2 |
| LR743457 | WL2 |
| LR743458 | WL2 |
| LR989885 | WL2 |
| LR989888 | WL2 |
| MF984337 | WL2 |
| MF984338 | WL2 |
| MF984339 | WL2 |
| MF984340 | WL2 |
| MF984341 | WL2 |
| MF984342 | WL2 |
| MF984343 | WL2 |
| MF984344 | WL2 |
| MF984345 | WL2 |
| MF984346 | WL2 |
| MF984347 | WL2 |
| MF984348 | WL2 |
| MF984349 | WL2 |
| MF984350 | WL2 |
| MF984351 | WL2 |
| MF984352 | WL2 |
| MH021189 | WL2 |
| MH244510 | WL2 |
| MH244511 | WL2 |
| MH244512 | WL2 |
| MH244513 | WL2 |
| MH549209 | WL2 |
| MH910045 | WL2 |
| MH924836 | WL2 |
| MH986055 | WL2 |
| MH986056 | WL2 |
| MK473443 | WL2 |
| MK947396 | WL2 |
| MK947397 | WL2 |
| MN480792 | WL2 |
| MN480793 | WL2 |
| MN480794 | WL2 |
| MN480795 | WL2 |
| MN481589 | WL2 |
| MN481590 | WL2 |
| MN481591 | WL2 |
| MN481592 | WL2 |
| MN481593 | WL2 |
| MN481594 | WL2 |
| MN481595 | WL2 |
| MN481596 | WL2 |
| MN481597 | WL2 |
| MN652878 | WL2 |
| MN652879 | WL2 |
| MN652880 | WL2 |
| MN794935 | WL2 |
| MN794937 | WL2 |
| MN794938 | WL2 |
| MN794939 | WL2 |
| MN939557 | WL2 |
| MN939558 | WL2 |
| MN939559 | WL2 |
| MN939560 | WL2 |
| MN939561 | WL2 |
| MN939562 | WL2 |
| MN939562 | WL2 |
| MN939564 | WL2 |
| MT341470 | WL2 |
| MT341471 | WL2 |
| MT341472 | WL2 |
| MT863560 | WL2 |
| MT863561 | WL2 |
| MW036634 | WL2 |
| MW142223 | WL2 |
| MW142224 | WL2 |
| MW142226 | WL2 |
| MW142227 | WL2 |
| AF196835 | TD01 / TD03 |
| AY277251 | TD01 / TD03 |
| AY532665 | TD01 / TD03 |
| AY701412 | TD01 / TD03 |
| AY701413 | TD01 / TD03 |
| AY765264 | TD01 / TD03 |
| DQ176636 | TD01 / TD03 |
| DQ256376 | TD01 / TD03 |
| DQ786573 | TD01 / TD03 |
| EF429197 | TD01 / TD03 |
| EF429198 | TD01 / TD03 |
| EF429199 | TD01 / TD03 |
| EF429200 | TD01 / TD03 |
| EU082200 | TD01 / TD03 |
| EU249803 | TD01 / TD03 |
| FJ159129 | TD01 / TD03 |
| FJ159130 | TD01 / TD03 |
| FJ159131 | TD01 / TD03 |
| FJ425721 | TD01 / TD03 |
| FJ483548 | TD01 / TD03 |
| FJ483549 | TD01 / TD03 |
| FJ766331 | TD01 / TD03 |
| FJ766332 | TD01 / TD03 |
| GQ379161 | TD01 / TD03 |
| GQ851602 | TD01 / TD03 |
| GQ851603 | TD01 / TD03 |
| GQ851604 | TD01 / TD03 |
| GQ851605 | TD01 / TD03 |
| GQ851606 | TD01 / TD03 |
| GQ851607 | TD01 / TD03 |
| GQ903680 | TD01 / TD03 |
| GU011992 | TD01 / TD03 |
| HM051416 | TD01 / TD03 |
| HM147822 | TD01 / TD03 |
| HM147823 | TD01 / TD03 |
| HM147824 | TD01 / TD03 |
| HM152775 | TD01 / TD03 |
| HQ537483 | TD01 / TD03 |
| JF707789 | TD01 / TD03 |
| JF719066 | TD01 / TD03 |
| JF719067 | TD01 / TD03 |
| JF719068 | TD01 / TD03 |
| JF719069 | TD01 / TD03 |
| JN393308 | TD01 / TD03 |
| JN858069 | TD01 / TD03 |
| JN858070 | TD01 / TD03 |
| JQ928174 | TD01 / TD03 |
| JQ928175 | TD01 / TD03 |
| JX041628 | TD01 / TD03 |
| JX041629 | TD01 / TD03 |
| JX041630 | TD01 / TD03 |
| JX041632 | TD01 / TD03 |
| JX041634 | TD01 / TD03 |
| JX123030 | TD01 / TD03 |
| JX123031 | TD01 / TD03 |
| JX442279 | TD01 / TD03 |
| JX556213 | TD01 / TD03 |
| KC407673 | TD01 / TD03 |
| KC496015 | TD01 / TD03 |
| KC496016 | TD01 / TD03 |
| KC601756 | TD01 / TD03 |
| KC954092 | TD01 / TD03 |
| KF179639 | TD01 / TD03 |
| KF179640 | TD01 / TD03 |
| KF234080 | TD01 / TD03 |
| KF647251 | TD01 / TD03 |
| KF647253 | TD01 / TD03 |
| KJ831223 | TD01 / TD03 |
| KJ883346 | TD01 / TD03 |
| KJ934710 | TD01 / TD03 |
| KM052152 | TD01 / TD03 |
| KM203861 | TD01 / TD03 |
| KM203862 | TD01 / TD03 |
| KM203863 | TD01 / TD03 |
| KP109692 | TD01 / TD03 |
| KP780837 | TD01 / TD03 |
| KP780838 | TD01 / TD03 |
| KP780839 | TD01 / TD03 |
| KP780840 | TD01 / TD03 |
| KT163243 | TD01 / TD03 |
| KT207791 | TD01 / TD03 |
| KT207792 | TD01 / TD03 |
| KT359349 | TD01 / TD03 |
| KT934796 | TD01 / TD03 |
| KT934797 | TD01 / TD03 |
| KT934798 | TD01 / TD03 |
| KT934799 | TD01 / TD03 |
| KT934800 | TD01 / TD03 |
| KT934801 | TD01 / TD03 |
| KT934802 | TD01 / TD03 |
| KT934803 | TD01 / TD03 |
| KU588135 | TD01 / TD03 |
| KY703854 | TD01 / TD03 |
| KY703855 | TD01 / TD03 |
| KY703856 | TD01 / TD03 |
| AF196835 | TD02 / TD03 |
| AF202541 | TD02 / TD03 |
| AF206518 | TD02 / TD03 |
| AF260967 | TD02 / TD03 |
| AF260968 | TD02 / TD03 |
| AF260969 | TD02 / TD03 |
| AF317203 | TD02 / TD03 |
| AF404753 | TD02 / TD03 |
| AF404754 | TD02 / TD03 |
| AF404755 | TD02 / TD03 |
| AF404756 | TD02 / TD03 |
| AF404757 | TD02 / TD03 |
| AF481864 | TD02 / TD03 |
| AF533540 | TD02 / TD03 |
| AJ965626 | TD02 / TD03 |
| AJ965628 | TD02 / TD03 |
| AM404308 | TD02 / TD03 |
| AY262283 | TD02 / TD03 |
| AY268132 | TD02 / TD03 |
| AY268133 | TD02 / TD03 |
| AY274504 | TD02 / TD03 |
| AY277252 | TD02 / TD03 |
| AY278441 | TD02 / TD03 |
| AY278442 | TD02 / TD03 |
| AY289214 | TD02 / TD03 |
| AY603654 | TD02 / TD03 |
| AY646354 | TD02 / TD03 |
| AY660002 | TD02 / TD03 |
| AY701412 | TD02 / TD03 |
| AY701413 | TD02 / TD03 |
| AY712945 | TD02 / TD03 |
| AY712946 | TD02 / TD03 |
| AY712947 | TD02 / TD03 |
| AY712948 | TD02 / TD03 |
| AY795965 | TD02 / TD03 |
| DQ005530 | TD02 / TD03 |
| DQ080051 | TD02 / TD03 |
| DQ080052 | TD02 / TD03 |
| DQ080053 | TD02 / TD03 |
| DQ080054 | TD02 / TD03 |
| DQ080055 | TD02 / TD03 |
| DQ080056 | TD02 / TD03 |
| DQ080057 | TD02 / TD03 |
| DQ080058 | TD02 / TD03 |
| DQ080059 | TD02 / TD03 |
| DQ080060 | TD02 / TD03 |
| DQ080061 | TD02 / TD03 |
| DQ080062 | TD02 / TD03 |
| DQ080063 | TD02 / TD03 |
| DQ080064 | TD02 / TD03 |
| DQ080065 | TD02 / TD03 |
| DQ080066 | TD02 / TD03 |
| DQ080067 | TD02 / TD03 |
| DQ080068 | TD02 / TD03 |
| DQ080069 | TD02 / TD03 |
| DQ080070 | TD02 / TD03 |
| DQ080071 | TD02 / TD03 |
| DQ080072 | TD02 / TD03 |
| DQ118127 | TD02 / TD03 |
| DQ164186 | TD02 / TD03 |
| DQ164187 | TD02 / TD03 |
| DQ164188 | TD02 / TD03 |
| DQ164189 | TD02 / TD03 |
| DQ164190 | TD02 / TD03 |
| DQ164191 | TD02 / TD03 |
| DQ164192 | TD02 / TD03 |
| DQ164193 | TD02 / TD03 |
| DQ164194 | TD02 / TD03 |
| DQ164195 | TD02 / TD03 |
| DQ164196 | TD02 / TD03 |
| DQ164197 | TD02 / TD03 |
| DQ164198 | TD02 / TD03 |
| DQ164199 | TD02 / TD03 |
| DQ164200 | TD02 / TD03 |
| DQ164201 | TD02 / TD03 |
| DQ164202 | TD02 / TD03 |
| DQ164203 | TD02 / TD03 |
| DQ164204 | TD02 / TD03 |
| DQ164205 | TD02 / TD03 |
| DQ164206 | TD02 / TD03 |
| DQ176637 | TD02 / TD03 |
| DQ211652 | TD02 / TD03 |
| DQ256376 | TD02 / TD03 |
| DQ374650 | TD02 / TD03 |
| DQ374651 | TD02 / TD03 |
| DQ374652 | TD02 / TD03 |
| DQ374653 | TD02 / TD03 |
| DQ377178 | TD02 / TD03 |
| DQ377179 | TD02 / TD03 |
| DQ377180 | TD02 / TD03 |
| DQ411029 | TD02 / TD03 |
| DQ411030 | TD02 / TD03 |
| DQ411031 | TD02 / TD03 |
| DQ411032 | TD02 / TD03 |
| DQ411033 | TD02 / TD03 |
| DQ411034 | TD02 / TD03 |
| DQ411035 | TD02 / TD03 |
| DQ431693 | TD02 / TD03 |
| DQ431694 | TD02 / TD03 |
| DQ431695 | TD02 / TD03 |
| DQ431696 | TD02 / TD03 |
| DQ431697 | TD02 / TD03 |
| DQ431698 | TD02 / TD03 |
| DQ431699 | TD02 / TD03 |
| DQ431700 | TD02 / TD03 |
| DQ431701 | TD02 / TD03 |
| DQ431702 | TD02 / TD03 |
| DQ431703 | TD02 / TD03 |
| DQ431704 | TD02 / TD03 |
| DQ431705 | TD02 / TD03 |
| DQ431706 | TD02 / TD03 |
| DQ431707 | TD02 / TD03 |
| DQ431708 | TD02 / TD03 |
| DQ431709 | TD02 / TD03 |
| DQ431710 | TD02 / TD03 |
| DQ431711 | TD02 / TD03 |
| DQ431712 | TD02 / TD03 |
| DQ666448 | TD02 / TD03 |
| DQ666449 | TD02 / TD03 |
| DQ666450 | TD02 / TD03 |
| DQ666451 | TD02 / TD03 |
| DQ666452 | TD02 / TD03 |
| DQ786572 | TD02 / TD03 |
| DQ786573 | TD02 / TD03 |
| EU249803 | TD02 / TD03 |
| FJ483548 | TD02 / TD03 |
| FJ483549 | TD02 / TD03 |
| FJ527738 | TD02 / TD03 |
| FJ766331 | TD02 / TD03 |
| FJ766332 | TD02 / TD03 |
| GQ379157 | TD02 / TD03 |
| GQ379158 | TD02 / TD03 |
| GQ379159 | TD02 / TD03 |
| GQ379160 | TD02 / TD03 |
| GQ379161 | TD02 / TD03 |
| GQ851602 | TD02 / TD03 |
| GQ851603 | TD02 / TD03 |
| GQ851604 | TD02 / TD03 |
| GQ851605 | TD02 / TD03 |
| GQ851606 | TD02 / TD03 |
| GQ851607 | TD02 / TD03 |
| GQ851608 | TD02 / TD03 |
| GU011992 | TD02 / TD03 |
| GU827998 | TD02 / TD03 |
| GU827999 | TD02 / TD03 |
| GU828000 | TD02 / TD03 |
| GU828001 | TD02 / TD03 |
| GU828002 | TD02 / TD03 |
| GU828003 | TD02 / TD03 |
| GU828004 | TD02 / TD03 |

**Table S4** Summary and comparison of parameter values from Beast analysis, parts A and B

(A) Result of marginal likelihood (log) estimation path sampling and stepping stone sampling methods for West Nile Virus Lineage 2 dataset using different coalescent models, and strict and uncorrelated relaxed log normal molecular clock models. (B) Calculation of best coalescent model and molecular clock model using Bayes factor. Bayes factor range 1-3 means hardly worth mentioning, 3-20 means postive support, 20-150 means strong support and >150 overwhelming support.

|  | Dataset | Sampling | Evolutionary Model | Strict | Uncorrelated relaxed log normal |
| --- | --- | --- | --- | --- | --- |
| **Part A** | European WNV Lineage 2 complete coding sequences | Stepping stone sampling | Constant | -29244.94581 | -29221.69928 |
|  |  |  | GMRF SkyRide | -29247.67687 | -29221.64373 |
|  |  |  | Bayesian SkyGrid | -29245.38443 | -29215.06113 |
|  |  | Path Sampling | Constant | -29248.94892 | -29218.79735 |
|  |  |  | GMRF SkyRide | -29243.10843 | -29216.66507 |
|  |  |  | Bayesian SkyGrid | -29242.2842 | -29211.11923 |

|  |  |  |  | Strict | | |  | Uncorrelated relaxed log normal | | |
| --- | --- | --- | --- | --- | --- | --- | --- | --- | --- | --- |
|  |  |  |  | Constant | GMRF SkyRide | Bayesian SkyGrid |  | Constant | GMRF SkyRide | Bayesian SkyGrid |
| **Part B** | Stepping stone sampling | Strict | Constant | 0.00 | -2.73 | -0.44 |  | 23.25 | 23.30 | 29.88 |
|  |  |  | GMRF SkyRide | 2.73 | 0.00 | 2.29 |  | 25.98 | 26.03 | 32.62 |
|  |  |  | Bayesian SkyGrid | 0.44 | -2.29 | 0.00 |  | 23.69 | 23.74 | 30.32 |
|  |  | Uncorrelated relaxed log normal | Constant | -23.25 | -25.98 | -23.69 |  | 0.00 | 0.06 | 6.64 |
|  |  |  | GMRF SkyRide | -23.30 | -26.03 | -23.74 |  | -0.06 | 0.00 | 6.58 |
|  |  |  | Bayesian SkyGrid | -29.88 | -32.62 | -30.32 |  | -6.64 | -6.58 | 0.00 |
|  | Path sampling | Strict | Constant | 0.00 | 5.84 | 6.66 |  | 30.15 | 32.28 | 37.83 |
|  |  |  | GMRF SkyRide | -5.84 | 0.00 | 0.82 |  | 24.31 | 26.44 | 31.99 |
|  |  |  | Bayesian SkyGrid | -6.66 | -0.82 | 0.00 |  | 23.49 | 25.62 | 31.16 |
|  |  | Uncorrelated relaxed log normal | Constant | -30.15 | -24.31 | -23.49 |  | 0.00 | 2.13 | 7.68 |
|  |  |  | GMRF SkyRide | -32.28 | -26.44 | -25.62 |  | -2.13 | 0.00 | 5.55 |
|  |  |  | Bayesian SkyGrid | -37.83 | -31.99 | -31.16 |  | -7.68 | -5.55 | 0.00 |
